# Dispersion Performances and Fluorescent Behaviors of Naphthalic Anhydride Doped in Poly(acrylic acid) Frameworks for pH-Sensitive Ibuprofen Delivery via Fractal Evolution

**DOI:** 10.3390/polym15030596

**Published:** 2023-01-24

**Authors:** Xueqing Cui, Xiaoli Wang, Xiaohuan Xu, Bang Xu, Jihong Sun, Shiyang Bai

**Affiliations:** Beijing Key Laboratory for Green Catalysis and Separation, Beijing University of Technology, Beijing 100124, China

**Keywords:** dispersion, drug delivery, poly(acrylic acid), 1,8-naphthalic anhydride, fluorescence, fractal, pH-sensitive

## Abstract

The pH-responsive fluorescent P(1,8-naphthalic anhydride (NA)-acrylic acid (AA)) matrix was successfully prepared by a doping method using poly(acrylic acid) (PAA) as a pH-sensitive polymer and NA as a fluorescent tracer. The fluorescent behaviors of the used NA dispersed in PAA frameworks were demonstrated based on fractal features combined with various characterizations, such as small-angle X-ray scattering (SAXS) patterns, photoluminescence (PL) spectra, scanning electron microscope (SEM) images, thermogravimetry (TG) profiles, Fourier transform infrared (FT-IR) spectroscopy, and time-resolved decays. The effects of NA-doping on the representative fluorescent P(NA-AA) were investigated, in which the fluorescent performance of the doped NA was emphasized. The results indicated that aggregated clusters of the doped NA were gradually serious with an increase in NA doping amount or extension of NA doping time, accompanied by an increase in mass fractal dimension (*D_m_*) values. Meanwhile, the doped NA presented stable fluorescent properties during the swelling–shrinking process of PAA. Ibuprofen (IBU) was used as a model drug, and fractal evolutions of the obtained P(NA-AA) along with the drug loading and releasing behaviors were evaluated via SAXS patterns, in which the drug-loaded P(NA-AA) presented surface fractal (*D_s_)* characteristics, while the *D_m_* value varied from 2.94 to 2.58 during sustained drug-release in pH 2.0, indicating occurrences of its structural transformation from dense to loose with extension of IBU-releasing time. Finally, the cytotoxicity and cellular uptake behaviors of the obtained P(NA-AA) were preliminarily explored. These demonstrations revealed that the resultant P(NA-AA) should be a potential intelligent-responsive drug carrier for targeted delivery.

## 1. Introduction

Over the past few decades, stimuli-sensitive polymers have demonstrated great potential for development of controlled and sustained drug delivery systems and reducing the side effects of drugs and, therefore, have received much attention for their biomedical applications [1,2,3,4,5]. They are more smartly responsive to very small changes associated with biological environment (e.g., pH, temperature, light, ionic strength, etc.) as compared to non-stimuli-responsive polymers [6]. As one of the most common polymers, poly(acrylic acid) (abbreviated as PAA) for pH-responsive behavior arouses constantly increasing interest. For example, Naficy et al. [7] fabricated poly(ethylene glycol) methyl ether methacrylates-PAA hydrogels. The results showed that their swelling ratio declined with a decrease in pH values, accompanied with transitioning to opaque from transparentthe primary reason being due to formation of dense hydrogen-bond clusters. Niu et al. [8] prepared a novel poly (propylene carbonate)-b-poly (acrylic acid) (PPC-b-PAA) pH-sensitive block copolymer, demonstrating excellent sensitivity to pH changes and high drug loading efficiency. Mackiewicz et al. [9] evaluated a kind of pH/redox stimuli-responsive PAA-based degradable microgel, not only displaying excellent drug-loading capacity but also showing better biocompatibility and lower biotoxicity. Nikravan et al. [10] prepared crosslinked PAA nanoparticles and found that the cumulative release rate of PAA at pH 1.2 was significantly greater than that at pH 7.4, presenting a pH-dependent performance with excellent controlled release behavior. The main reason may be that the forces generated by shrinkage under an acidic environment drive drug molecules outside the nanoparticles. Similar phenomena were also demonstrated by Wei et al. [11] as prepared hydrogels (salecan and poly(N,N-diethylacrylamide-co-methacrylic acid) semi-interpenetrating polymer networks) presented higher drug-release rates under acidic conditions, the main reason being that the carboxyl group was protonated under acidic conditions, leading to shrinking of the hydrogels, which is conductive to release of the loaded drug. As demonstrated by Dadfar et al. [12], a high cumulative release rate appeared under acidic conditions via synthesized hydrogels (N-isopropylacrylamide and Nethylmaleamic acid and sodium alginate polymer semi-interpenetrating polymer networks) due to hydrostatic pressure caused by shrinkage. These examples indicate that the PAA-based copolymer, as a drug carrier, possessed excellent pH-response behavior in the drug delivery system.

Fluorescent molecules have been widely used to label polymer-carriers for tracking and detecting drug delivery at the correct location and exact time in the body. Liang et al. [13] prepared a new fluorescent polymeric nanoparticle using PAA and naphthalimide compound as precursors, showing low cytotoxicity, water solubility, and good fluorescence properties. Liang et al. [14] thereafter found that the naphthalimide-grafted PAA possessed excellent responsibility for pH variation and strong fluorescence activity. Cai et al. [15] also elucidated that the PAA-surface-crosslinked fluorescein copolymers had both strong fluorescence and a suitable nanoparticle structure, which can be used for in vitro imaging of cancer cells and in vivo long-life imaging of in situ tumors.

In our previous explorations with amine-modified 1,8-Naphthalic anhydride (NA) as a fluorescent tracer and PAA as a pH-sensitive polymer, pH-sensitive fluorescent polymers were obtained via polymerization. Meanwhile, using vinyl-modified bimodal mesopores SiO_2_ (abbreviated as BMMs) as a core and pH-sensitive fluorescent polymers as a shell, pH-responsive fluorescent hybrid nanoparticles (P@BMMs) with core-shell structures were successfully prepared. The results revealed that the fluorescence intensity of the resultant P@BMMs nanoparticles could achieve a good fluorescence labeling effect for potential application in tracing drug sustained/controlled delivery fields [16]. However, the fluorescent performances are uncertain in the drug delivery process due to unclear understanding of the dispersion behaviors and agglomeration properties of organic fluorescent molecules in the drug carrier. However, the literature is rarely reported. As demonstrated by Ghosh et al. [17], the absorption, steady-state, and time-resolved fluorescent spectra of NA in various aprotic solvents with different polarities indicated that the fluorescent intensity strongly depended on the used solvent physicochemical properties. Tarai et al. [18] systematically investigated the relationships between position of emission peak and concentration. They found that the position of the emission peak of the synchronous fluorescence spectrum redshifted with an increase in the concentration regardless of the mono-fluorescence system or multi-fluorescence system. Yu et al. [19] prepared a series of deep-red/NIR emissive AIEgens and further explored the influences of the interactions between luminogens and solvent molecules in dilute solution or their interactions in solid fluorescence. These results indicated that strong π–π and D–A interactions easily caused redshift in emission.

As is well known, small-angle X-ray scattering (SAXS) technology has become one of the effective methods to explore structural inhomogeneities of copolymers in recent years, which can provide new information about their microstructures, including their fractal features, irregular geometric shapes, and related morphology variations. For example, Cong et al. [20] demonstrated segment-level inhomogeneity of block copolymer networks (PNIPAM-block-PAA) with varying PAA content by SAXS patterns. Zhao et al. [21] found that, when acryloyloxyethyltrimethyl ammonium chloride was introduced into networks of poly(sodium 2-acrylamide-2-methylpropanesulfonate) double-network hydrogels and co-polymerized with acrylamide monomer, the SAXS profiles of the obtained hydrogels showed prominent upturns toward the low *q* region. This variance showed that the hydrogels had developed large-scale inhomogeneities. Dispersion of organic fluorescent molecules, swelling–shrinking behaviors, drug delivery performances, and fractal evolutions in the polymer chain, however, are rarely reported in detail but have presently become the focus of current works. For these reasons, pH-responsive fluorescent polymer P(NA-AA) was prepared by doping method using PAA as a pH-sensitive polymer and NA as a fluorescence tracer. Herein, SEM images were used to analyze the apparent morphologies and microstructure characteristics of these crosslinking networks. The dispersion behaviors of NA in PAA networks and the stability during the swelling–shrinking process were demonstrated by photoluminescent spectroscopy (PL). Meanwhile, ibuprofen (IBU) was used as a model drug, the luminescent mechanism between dispersion behavior of NA and its agglomeration in the PAA networks was further clarified by using time-resolved fluorescence profiles and SAXS patterns, and the drug loading/releasing performances were evaluated at pH 2.0 and pH 7.4 at 37 °C, along with characterizations of the NA structures and properties. This study is also designed to investigate their cytotoxicity in cells and cellular uptake behaviors.

## 2. Materials and Methods

### 2.1. Materials

NA (98% of purity), IBU (98%), azodiisobutyronitrile (AIBN, 98%), N, N methylenebisacrylamide (MBA, 99%), and acetonitrile (99%) were purchased from Aladdin company. AA (99%) and ethanol (99.7%) were provided from Fuchen (Tianjin) Chemical Reagent Co., Ltd. (Tianjin, China), and sodium dihydrogen phosphate disodium hydrogen phosphate and phosphoric acid were supplied by Beijing chemical works. The phosphate-buffered saline (PBS) at pH 2.0 and pH 7.4, respectively, were prepared in proportion ratio of the sodium dihydrogen phosphate, disodium hydrogen phosphate, and phosphoric acid. The specific preparation method is as follows:

Preparation of PBS at pH 2.0: Solution A: 1.66 mL of phosphoric acid was added to deionized water (up to 1000 mL) and then shaken well. Solution B: 7.16 g of disodium hydrogen phosphate was added to deionized water (up to 1000 mL) and then shaken well. After that, 725 mL of Solution A and 275 mL of Solution B 275 mL were mixed and then shaken well.

Preparation of PBS at pH 7.4: Solution A: 7.16 g of disodium hydrogen phosphate was added to deionized water (up to 1000 mL) and then shaken well. Solution B: 3.12 g of sodium dihydrogen phosphate was added to deionized water (up to 1000 mL) and then shaken well. After that, 810 mL of Solution A and 190 mL of Solution B were mixed and then shaken well.

Deionized water with resistivity of 18.25 MΩ cm was obtained by ZHIANG-Best Water Purifier in our lab. Human cervical cancer cells (HeLa) were obtained from Saiqi (Shanghai) Biological Engineering Co., Ltd. (Shanghai, China), and glutamine, Dulbecco’s modified Eagle’s medium (DMEM) cell culture media, fetal bovine serum (FBS), penicillin/streptomycin, 0.25% trypsin/EDTA solution, and PBS were obtained from HyClone. Cell Counting Kit-8 (CCK-8) was supplied by Dojindo Molecular Technologies (Dojindo, Japan).

### 2.2. P(NA-AA) Prepared by One-Step Method

The detailed preparation of P(NA-PAA) by adding NA at different times was as follows: AA (5 mL) was dispersed in anhydrous ethanol (20 mL) using a 100 mL 3-neck-flask equipped with a condenser. The reaction mixture was stirred and kept under a nitrogen atmosphere for about 30 min to remove oxygen, and then cross-linking agent MBA (250 mg) and initiator AIBN (50 mg) were added to initiate polymerization. NA (0.02625 g; the mass ratio of NA to AA was 0.5%) was added at a certain time. The reaction was carried out under the protection of N_2_ at 70 °C and refluxed for 7 h. The product obtained was purified by ethanol three times, dried, and then marked as P(NA-AA)-x (x is the time when NA is added, and selected as 0, 0.2, 0.5, 1, 3, and 7, respectively).

The detailed preparation of P(NA-PAA) by additive various amounts of NA was as follows: AA (5 mL) was dispersed in anhydrous ethanol (20 mL) using a 100 mL 3-neck-flask equipped with a condenser. The reaction mixture was stirred and kept under a nitrogen atmosphere for about 30 min to remove oxygen, and then cross-linking agent MBA (250 mg) and initiator AIBN (50 mg) were added to initiate polymerization. A certain amount of NA was added at 0, 1, and 7 h, respectively. The reaction was carried out under the protection of N_2_ at 70 °C and refluxed for 7 h. The product obtained was purified by ethanol three times, dried, and then marked as P(NA-AA)-x-y (y is the value of the mass ratio of NA to AA, and selected as 0.05, 0.1, 0.5, 1, 3, 5, 7, and 10, respectively).

### 2.3. NA-PAA Prepared by Physical Mixed Method

The NA-PAA was prepared by physical grinding of previously prepared PAA and NA. A certain amount of NA and PAA were ground using mortar and pestle for 1 h, then marked as NA-PAA-z (z is the value of the mass ratio of NA to PAA, and selected as 0.05, 0.1, 0.5, 1, 3, 5, 7, and 10, respectively).

### 2.4. Swelling Behaviors

To investigate the swelling behavior of the resultant samples in different pH values, the samples were immersed in PBS (40 mL, pH 2.0 or pH 7.4) at room temperature. The gravimetric method was used to determine the hydrogel’s water intake. The samples (about 250 mg) were placed in the solutions (40 mL) and removed at regular time intervals, removed any excess water superficially with filter paper, weighed, and then placed back into the buffer. Measurements were continued until a constant weight was reached for each sample. The tests were conducted in triplicate. The *swelling ratio* (*SR*) values were computed according to the shown Equation (1):

(1) $$SR\;\%=\frac{W_{t}{\;-\;W}_{d}}{W_{d}}\;\times \;100\%$$

where *W_d_* is the weight of the freeze-dried sample, *W_t_* is the weight of swollen polymer at time *t*.

### 2.5. IBU Loading

The IBU was loaded into the fluorescent polymers by incubating the P(NA-AA)-x-y in the solution of IBU of concentration (40 mg/mL). 300 mg of P(NA-AA)-x-y was incubated in 30 mL of IBU solution at room temperature (25 °C) and stirred slowly in a mechanical shaker for 48 h. Finally, the I/P(NA-AA)-x-y was obtained by filtered separation and washed twice with ethanol. To measure the mass of IBU loaded into P(NA-AA)-x-y, the filtrate was diluted with ethanol up to 100 mL. The drug loading content (Equation (2)) and loading efficiency (Equation (3)) were obtained by HPLC (Agilent 1200 Series Waldborn, Germany) with a standard calibration curve (as shown in Figure S1 of the Electronic Supporting Information (ESI) section).

(2) $$LC\;\%=\frac{M_{0}-{\;M}_{1}}{M_{2}}\;\times \;100\%$$

(3) $$EE\;\%=\frac{M_{0}-{\;M}_{1}}{M_{0}}\;\times \;100\%$$

where *M*_0_ is the total weight of IBU initially, *M*_1_ is the weight of IBU remaining in the soaked medium after equilibrium, *M*_2_ is the weight of I/P(NA-AA)-x-y following lyophilization.

### 2.6. In Vitro Release of IBU

In vitro drug release experiments of IBU-loaded samples were investigated in PBS buffer with different pH values (pH 7.4 and 2.0). The lyophilized IBU-loaded samples (about 25 mg) were put into a dialysis bag (cut-off 3500 Da), which was sealed and immersed into 20 mL PBS at 37 ± 0.5 °C with continuous shaking at 100 rpm. 1 mL of dialysate was withdrawn at defined time intervals and an equal volume of fresh dialysate was replenished. The pH value of the drug-releasing system was monitored with a pH meter, showing a pH value of around 2.0 (or 7.4). The release experiments were conducted in triplicate. To calculate the released amount of drug, a calibration curve of IBU in PBS was taken (as shown in Figure S2 of the ESI section). The collected sample fractions were then used to scrutinize the amount of IBU using UV–vis spectrophotometer at a preset wavelength value of 272 nm. The cumulative release rate can be calculated by following Equation (4):

(4) $$\mathrm{Cumulative}\;\mathrm{Release}\;\mathrm{rate}\;\%=\sum_{t=0}^{t}\frac{C_{t\;}\times \;V}{M}\;\times \;100\%$$

where *C_t_* (mg/mL) is the apparent concentration at time t, *V* is the volume of total volume of release fluids (mL), *M* is the IBU loading amount.

### 2.7. In Vitro Cytotoxicity Experiments

In vitro cytotoxicity of the carrier (PAA, P(NA-AA)−0−0.1, P(NA-AA)−0−1, P(NA-AA)−0−10, NA) was evaluated by the CCK-8 assay. HeLa cells were used as the tumor cell models. DMEM was used as the control group. Experiments of each carrier were conducted in triplicate. The detailed procedure: HeLa cells were cultured in DMEM at 37 °C in an incubator with saturated humidity and 5% CO_2_. The cells were transferred from the culture bottle to 96-well plates at a density of 8 × 10^3^ cells per well. After being cultured for 24 h, the medium was sucked out and 200 µL of the culture medium contained different concentrations (1, 5, 10, 20, 50, 100 µg/mL) of the carrier.

After being cultured for 48 h, the culture medium was removed and the adhered cells were washed with PBS solution. 100 µL of the CCK-8 solution (10% CCK-8) was then added to each well in the dark. After 1 h of continuous culture in the incubator, the optical density (OD) value at 450 nm was determined by a microplate reader. Cell viability values were obtained after data treatment. The cell viability values were calculated by using Equation (5) as follows:

(5) $$\mathrm{Cell}\;\mathrm{Viability}\;(\%)=\frac{{\mathrm{OD}}_{\mathrm{Sample}\;}-{\;\mathrm{OD}}_{\mathrm{Blank}}}{{\mathrm{OD}}_{\mathrm{Control}}-{\;\mathrm{OD}}_{\mathrm{Blank}}}\;\times \;100\%$$

### 2.8. In Vitro Cellular Uptake Study

HeLa cells were seeded on glass culture dishes (3 × 10^4^/well, 2 mL cell suspension) and incubated overnight at 37 °C, 5% CO_2_. Then, the cells were washed twice with PBS and incubated at 37 °C with DMEM containing 20 µg/mL P(NA-AA)−0−0.1 for 6, 24, and 48 h. The cells were washed with PBS before being examined under confocal laser scanning microscope (CLSM, A1Rsi, Nikon, Kyoto, Japan). The excitation source was a 405 nm diode laser for compounds.

### 2.9. Characterizations

The PL spectrum was carried out on F7000 luminescence spectrometer equipped with a Xenon lamp of 150 W as an excitation light source. The emission spectra were collected under excitation 300 nm with a bandwidth of 2.5 nm at room temperature. The SEM images were obtained with a Hitachi S-4800 field-emission scanning electron microscope at an acceleration voltage of 5.0 kV. Using Image J (National Institutes of Health) technique, the network and morphology of polymer were evaluated. High-performance liquid chromatography (HPLC) of Agilent Technologies 1200 series was used for analysis of samples, and chromatographic column was Extend-C18. UV–vis absorbance spectra were measured using a UV–vis spectrophotometer (Shimadzu UV-2600 Kyoto, Japan). Time-resolved decay of samples obtained using FLS-1000 analytical instruments. TG-DSC experiments were performed with a thermogravimetric analyzer (PerkinElmer STA 8000) in a temperature range of 30–900 °C at a heating rate of 10 °C·min^−1^ with an air flow rate of 20 mL·min^−1^. FT-IR spectrum of obtained samples was recorded using IR Prestige-21 in the wavenumber range of 400–4000 cm^−1^. The cells were counted using a cell counting chamber under the inverted microscope (XDS-1B, COIC). Absorbance of plates was measured using a microplate reader (Thermo Scientific, Waltham, MA, USA) at 450 nm. Cell uptake and P(NA-AA)−0−0.1 distribution in cells were observed using a confocal laser scanning microscope (CLSM, A1Rsi, Nikon). The pH value was monitored with a pH meter (S220 SevenCompact meter, METTLER-TOLEDO, Shanghai, China). SAXS patterns were measured at Beamline 1W2A at the Beijing Synchrotron Radiation Facility [22]. The powder samples were supported on adhesive tape for measurement, which was used for background correction for the samples [23]. The sample-to-detector distance for SAXS was 1.58 m, calibrated with the diffraction ring of a standard sample. The scattering vector magnitude *q* varied from 0.09 to 3.05 nm^−1^. Fit2D software was used to transform the two-dimensional SAXS data into one-dimensional intensity *I*(*q*) as a function of *q* (*q = 4πsinθ*/*λ*) before being further processed using the S program [24]. It has been reported that the mass fractal dimension (*D_m_*) value can be obtained in the range of 0 < α < 3, *D_m_* = α while the surface fractal dimension (*D_s_*) value in the range of 3 < α < 4, *D_s_* = 6 − α (*D_s_* ≠ 3) [25].

## 3. Results and Discussion

### 3.1. The Fluorescent Characteristics of the P(NA-AA)

Using acetonitrile as a solvent and NA as a solute, the concentration dependence of the emission spectral shift was investigated over a concentration range from 1 × 10^−1^ to 1 × 10^−4^ M to determine the relationships between the dispersion state of NA in the solvent and its emission peak position. Their emission spectra are shown in Figure S3A. As can be seen in Figure S3A-a, the referred acetonitrile presented no emission peak in the range of 350–500 nm, while solid NA (Figure S3A-j), as an aggregated state, showed an emission peak position of 450 nm. Obviously, the peaks located at 392.5 nm (1.0 × 10^−1^ M, as shown in Figure S3A-i), 384.5 nm (5.0 × 10^−2^ M, as shown in Figure S3A-h), 381.9 nm (1.0 × 10^−2^ M, as shown in Figure S3A-g), 379.5 nm (8.3 × 10^−3^ M, as shown in Figure S3A-f), 376.8 nm (5.0 × 10^−3^ M, as shown in Figure S3A-e), and 376.0 nm (3.3 × 10^−3^ M, as shown in Figure S3A-d) belonged to the emission wavelength of the transition state between the monodisperse state and aggregated state of NA. Meanwhile, the wavelength gradually blue-shifted with decreases in NA concentration in acetonitrile, showing a downward trend of the NA aggregation; the probable reason is that the dilute effect of acetonitrile on the monomers of NA is obvious. However, when the NA concentration in acetonitrile was around 2.5 × 10^−4^–1.0 × 10^−4^ M, the peak position of the emission spectra remained unaltered at about 375 nm (Figure S3A-b and -c), speculating the existence of NA in the acetonitrile solutions as a monomer. These results indicated that the emission differences at different aggregation states can be utilized as indicators to measure fluorescence dispersion state. Similar observations were also described previously by some reports [18,26]. As demonstrated by Tarai et al. [18], when the R6G group was in a very dilute solution, the position of the emission peak remained almost invariant with the concentration until the concentration reached 1 × 10^−5^ M and the position of the emission peak red-shifted. Li et al. [26] found that the monodisperse state of 2-[3-(triethoxysilyl) propyl-1H-Benz [de] isoquinoline-1,3(2H)-dione] appeared in ethanol when its concentration was less than 1 × 10^−7^ mol/L and the monomer emission peak was at 390 nm.

**Figure 1:**
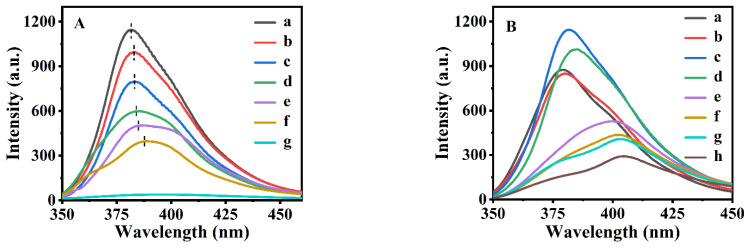
Emission spectra of P(NA-AA)-x-0.5 doped with NA at different times (**A**) and P(NA-AA)−0−y (B) with various doped amount of NA (**B**). (**A**): (a) P(NA-AA)-0-0.5P(NA-AA)−0−0.5, (b) P(NA-AA)−0.2−0.5, (c) P(NA-AA)−0.5−0.5, (d) P(NA-AA)−1−0.5, (e) P(NA-AA)−3−0.5, (f) P(NA-AA)−7−0.5, and (g) PAA. (**B**): (a) P(NA-AA)−0−0.05, (b) P(NA-AA)−0−0.1, (c) P(NA-AA)−0−0.5, (d) P(NA-AA)−0−1, (e) P(NA-AA)−0−3, (f) P(NA-AA)−0−5, (g) P(NA-AA)−0−7, and (h) P(NA-AA)−0−10.

The emission spectra of the synthesized P(NA-AA)-x-0.5 are shown in Figure 1A. As can be seen in Figure 1A-g, PAA presented no emission peak in the 350–460 nm range, while the synthesized P(NA-AA)-x-0.5 (x = 0, 0.2, 0.5, 1, 3, and 7) showed superposition of two peaks of around 380 and 400 nm, indicating the appearances of two aggregation states of the added NA in the PAA networks. Combined with the results of the monodisperse experiments (as shown in Figure S3B), it can be speculated that the additive NA clusters represented by the latter peak (about 400 nm) were more aggregative than that represented by the former peak (about 380 nm). The larger the peak area was, the larger its proportion was; accordingly, we noticed that the low aggregated NA was still dominant, although the ratios of the peak (380 nm) area to those (400 nm) of P(NA-AA)-0-0.5, P(NA-AA)-0.2-0.5, P(NA-AA)-0.5-0.5, P(NA-AA)-1-0.5, P(NA-AA)-3-0.5, and P(NA-AA)-7-0.5 were 1.18, 1.15, 1.13, 1.12, 1.09, and 1.05, respectively, demonstrating decreasing inclinations with extension of NA additive time. Therefore, the results and discussion were based on the low aggregated NA in the following section.

Aside from the decrease in the fluorescence intensity of the emission peak with the extension of NA additive time, we observed that the position of the emission peak of the synthesized P(NA-AA)-x-0.5 was red-shifted from 382.0 nm for P(NA-AA)-0-0.5 (Figure 1A-a), to 382.8 nm for P(NA-AA)-0.2-0.5 (Figure 1A-b), 383.8 nm for P(NA-AA)-0.5-0.5 (Figure 1A-c), 384.3 nm for P(NA-AA)-1-0.5 (Figure 1A-d), 385.2 nm for P(NA-AA)-3-0.5 (Figure 1A-e), and 388.2 nm for P(NA-AA)-7-0.5 (Figure 1A-f), respectively. These phenomena suggest that prolongation of NA additive time was conducive to enhancement of the aggregation trend of the used NA dispersed in PAA.

To further investigate the influences of the additive doped NA amounts on their dispersion performances, P(NA-AA)−0−y, P(NA-AA)−1−y, and P(NA-AA)−7−y with various doped NA amounts in PAA were selected. As illustrated in Figure 1B, the emission spectra of the synthesized P(NA-AA)−0−y presented almost the same as the profiles in Figure 1A, appearing with two peaks centered at 380 and 400 nm. However, when the NA-doped amount was less than or equal to 1% (as presented in Figure 1B-a–B-d), the peak (380 nm) area of low aggregations was larger than that (400 nm) of high aggregations, implying main existences of low NA-aggregated species; when more than 1% (as shown in Figure 1B-e–B-h), the opposite was present. Meanwhile, the red-shiftiness of their emission peak position occurred with an increase in NA-doped amount. The emission spectra of other samples also presented the same profiles, as shown in Figure S4A,B of the ESI section.

Additionally, the emission spectra of NA-PAA-z obtained by the physical mixing method are presented in Figure S4C. As can be seen, appearance of two or more emission peaks, such as 380, 400, and 430 nm, indicated the doped NA in PAA aggregated seriously with uneven distribution compared with that obtained by the one-step method. As presented in Figure S4D, we also found that the emission peak position of the mixed NA-PAA (Figure S4D-d) was red-shifted with an increase in doped NA amount, particularly moving toward the longer wavelength regions compared to the one-step-doped samples (Figure S4D-a–D-c).

**Figure 2:**
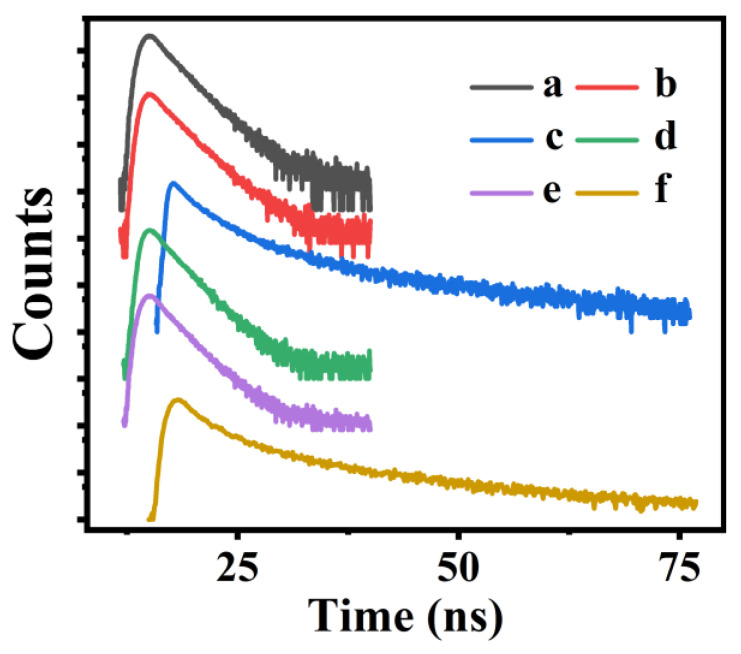
Time-resolved decay profiles and corresponding decayed time (insert) of (a) P(NA-AA)−0−0.1, (b) P(NA-AA)−0−1, (c) P(NA-AA)−0−10, (d) P(NA-AA)−1−0.1, (e) P(NA-AA)−7−0.1, and (f) NA.

The fluorescence decay profiles of NA and P(NA-AA)-x-y are shown in Figure 2. As can be seen in Figure 2f, the fluorescence decay of pure NA was bi-exponential, with lifetimes of 2.615 ns and 14.780 ns. After doping in PAA, the lifetimes reduced to 2.389 and 11.350 ns for P(NA-AA)−0−10 (Figure 2c), but 2.341 ns for P(NA-AA)−0−1 (Figure 2b) and 2.102 ns for P(NA-AA)−0−0.1 (Figure 2a), showing a declining tendency with a decrease in doped NA amount. One of the possible reasons may be due to decreases in electron cloud density in the NA naphthalene ring originating from existence of electron absorbing groups (-COOH) in the PAA frameworks [27].

The fluorescence decay of P(NA-AA)−0−0.1, P(NA-AA)−1−0.1, and P(NA-AA)−7−0.1 (as shown in Figure 2a, d, and e) was mono-exponential, with lifetimes of 2.102, 2.126, and 2.203 ns. The probable reasons may be due to aggregation of the doped NA with the time extension of the doped NA. Similar phenomena were also reported by Hu’s research group [28]: the fluorescence lifetime was gradually extended from 0.4 to 6.3 ns with enhancement of the aggregation state of the used thioxanthone.

### 3.2. The Structure Features of the P(NA-AA)

**Figure 3:**
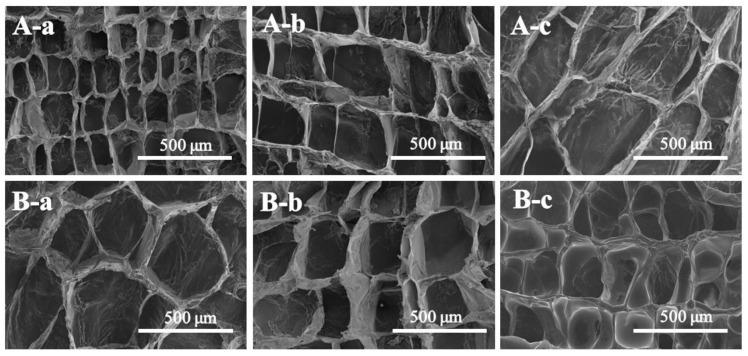
SEM images of P(NA-AA)−0−0.1 in PBS at pH 7.4 (**A**) and pH 2.0 (**B**) at 37 °C with various swelling/shrinking times. (a) 1 h, (b) 5 h, and (c) 24 h.

The micropore morphological features of the P(NA-AA)−0−0.1, as presented in Figure 3, were taken for the lyophilized samples after swelling at different time intervals. Indeed, all the freeze-dried samples are highly porous. The statistical analyses revealed that their average pore sizes in PBS at pH 7.4 increased substantially with an increase in swelling time, showing 183.7, 364.8, and 442.4 µm at a swelling time of 1, 5, and 24 h, as displayed in Figure 3A-a–A-c, respectively. However, as demonstrated in Figure 3B-a–B-c, their average pore size at pH 2.0 steadily dropped to 235.7 m throughout swelling time (24 h). These findings supported the swelling–shrinking characteristics of P(NA-AA)−0−0.1.

**Figure 4:**
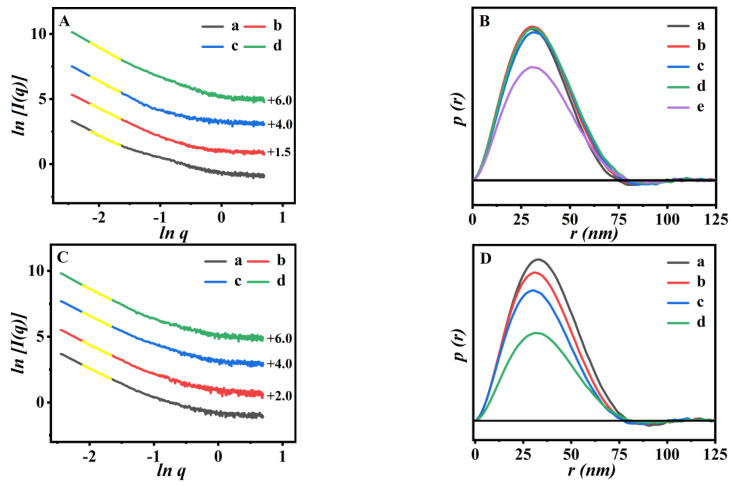
Shifted scattering curves (**A**) with their fractal values (insert), and PDDF profiles (**B**) of (a) PAA, (b) P(NA-AA)−0−0.1, (c) P(NA-AA)−0−1, and (e) P(NA-AA)−0−10. Shifted scattering patterns (**C**) with their fractal values (insert), and PDDF profiles (**D**) of (a) P(NA-AA)−0−0.1, (b) P(NA-AA)−1−0.1, (c) P(NA-AA)-3-0.1, and (d) P(NA-AA)−7−0.1. Notes: (1) the linear ranges in Figure 4A,C with a higher coefficient (*R^2^* > 0.99) were −2.10 < ln*q* < −1.67. (2) Figure 4A,C shows the offset values on the right *y*-axis.

The shifted scattering patterns of the obtained P(NA-AA)−0−y and their pair distance distribution function (PDDF) profiles are shown in Figure 4. In Figure 4A, better linear relations (*R^2^* > 0.99) evident in the low-*q* range of the shifted scattering patterns were present, with slopes of −2.25, −2.35, −2.49, and −2.65, respectively, indicating that the *D_m_* value increases from 2.25 to 2.65. These findings suggest that the pores in the prepared P(NA-AA)−0−y became dense with an increase in NA-doped amount, which may be related to aggregation of the NA clusters, in close agreement with the PL observations in Figure 1B.

The PDDF profiles of P(NA-AA)−0−y are shown in Figure 4B. Indeed, P(NA-AA)−0−y exhibited a bell-shaped function (*p*(*r*)~*r*) with a narrow distribution, assuming the appearance of the regular and aggregated particles with a shaped ellipsoid [29].

Based on the Guinier equation [30], the Guinier radius values of gyration (*R_g_*) can be computed from SAXS data in the low-*q* regions as follows:

(6) $$I_{G}\left(q\right){=I}_{G}\left(0\right)\exp(-\frac{q^{2}R_{g}^{2}}{3})$$

where *I_G_*(0) is the asymptotic value of the Guinier intensity when the scattering vector (*q*) is close to 0. Herein, the *R_g_* values should be linked to the aggregated size of the prepared P(NA-AA)−0−y stemming from their cross-link domains and their maximum diameter (*D_max_*) [31,32].

Accordingly, the *D_max_* values of PAA, P(NA-AA)−0−0.1, P(NA-AA)−0−1, and P(NA-AA)−0−10 were 74.6, 76.6, 77.9, and 81.1 nm, exhibiting that *D_max_* values gradually increased with an increase in the NA-doped amount. The variation trends of the *R_g_* values, presenting 25.6, 26.2, 26.7, and 27.6 nm for PAA, P(NA-AA)−0−0.1, −1, and −10, respectively, were comparable to those of *D_max_* values. The most probable explanations are related to remarkable aggregation of the doped NA with an increase in the doped NA amount. The normalized Kratky plots of P(NA-AA)−0−y were specifically presented in Figure S5A in the ESI section. Their signal intensities increased gradually in larger *q*-range (*q* > 1) with an increase in the NA-doped amount, indicating that the conformation of P(NA-AA)−0−y appears to be abundant and dominated by partially unfolded states, similar to our previous report [33].

Subsequently, the shifted scattering patterns of the PDDF profiles of the obtained P(NA-AA)-x-0.1 with their fractal values are shown in Figure 4C,D. Figure 4C demonstrates the yellow line segments for each scattering pattern in the low-*q* range with an excellent correlation (*R^2^* > 0.99). In detail, the *D_m_* values of P(NA-AA)-x-0.1 presented increased tendencies from 2.37 for P(NA-AA)−0−0.1 to 2.46 for P(NA-AA)−7−0.1 with extension of NA additive time. These results indicated that the pores in the prepared P(NA-AA)-x-0.1 became dense with extension of NA additive time. The probable reasons are related to the more aggregative NA clusters, in close agreement with the PL observations in Figure 1A.

The PDDF profiles of the P(NA-AA)-x-0.1 are shown in Figure 4D. Indeed, P(NA-AA)-x-0.1 exhibited symmetrical bell-shaped profiles, showing that the P(NA-AA)-x-0.1 were ellipsoidal, similar to P(NA-AA)−0−y (as shown in Figure 4B), while the impacts of NA additive time on *R_g_* values of P(NA-AA)-x-0.1 were unobvious. Meanwhile, Figure S5B presented the Kratky plots of P(NA-AA)-x-0.1, similar to P(NA-AA)−0−y.

As shown in Figure S6A, the weight loss of 30–200 °C in all samples was associated with removal of the adsorbed H_2_O. The weight loss of about 23.9 wt% for PAA (as shown in Figure S6A-a) corresponded to the decarboxylation process during the temperature range of 200–300 °C. Subsequently, weight loss of about 57.7 wt% happened between 300–500 °C due to decompositions of the PAA [34]. As shown in Figure S6A-e, the occurrences in the temperature periods of 200–340 °C were attributed to thermal decomposition of NA with weight loss of about 97.2 wt%. Comparably, Figure S6A-b–A-d profiled that the weight change between 200 and 300 °C was due to decomposition of NA and decarboxylation of PAA. Correspondingly, Figure S6B-b–B-d presented that the maximum decomposition rate based on the DTG curves gradually moved to high temperature with increasing NA-doped amount, indicating that the thermal decomposition temperature increased with aggregation of NA clusters. Sovizi’s group [35] also reported similar observations that the decomposition temperature of nitrocellulose increased with its particle size.

Figure S7 shows the FT-IR spectra of PAA, NA, and P(NA-AA) copolymers. As shown in Figure S7a, the absorption peak of PAA at 1701 cm^−1^ was ascribed to the stretching vibration of C=O in the -COOH group [36], while the bands at 1541 and 1453 cm^−1^ could be attributed to asymmetric and symmetric stretching vibrations of COO^-^ anion groups, respectively [37]. Especially, no peak at 1637 cm^−1^ was observed, indicating the absence of C=C group and successful synthesis of the PAA [38]. Additionally, the extra peaks located at 772, 841, and 1016 cm^−1^ (as shown in Figure S7b–d) should be ascribed to the characteristic absorption bands of NA [39], which may imply that the NA was doped into the PAA skeletons.

### 3.3. The Swelling Behaviors

**Figure 5:**
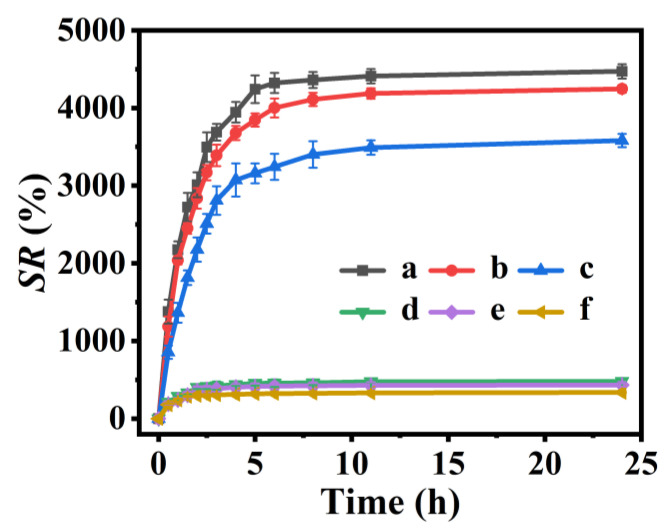
SR profiles of P(NA-AA)−0−y at 37.0 ℃ under pH 7.4 (y = 0.1 (a), 1.0 (b), and 10.0 (c)) and 2.0 (y = 0.1 (d), 1.0 (e), and 10.0 (f)).

The P(NA-AA)-x-y were used as the pH-sensitive carriers, and their drug delivery performances were evaluated by investigating their swelling behavior in solutions with various pH values at 37.0 °C. As illustrated in Figure 5, the *swelling ratio* values of P(NA-AA)−0−0.1, P(NA-AA)−0−1, and P(NA-AA)−0−10 were about 4472, 4246, and 3580% at pH 7.4 (Figure 5A-a–A-c) but 479, 432, and 337% at pH 2.0 (Figure 5A-d–A-f), respectively. Similar results were also observed in other samples (as shown in Figure S8): the *SR* values of P(NA-AA)−1−0.1, P(NA-AA)−1−1, and P(NA-AA)−1−1P(NA-AA)−1−10 were about 4199, 3781, and 3276% at pH 7.4 (Figure S8A-a–A-c) but 422, 369, and 300% at pH 2.0 (Figure S8A-d–A-f), respectively. The *SR* values of P(NA-AA)−7−0.1, P(NA-AA)−7−1, and P(NA-AA)−7−10 were about 3906, 3638, and 2958% at pH 7.4 (Figure S8B-a–B-c) but 420, 344, and 293% at pH 2.0 (Figure S8B-d–B-f), respectively. These observations revealed that the *SR* values at pH 7.4 or 2.0 slightly decreased with an increase in the NA-doped amount or extension of the NA-doped time, suggesting that the doped NA has little influence on the swelling performances. Obviously, these excellent pH-sensitive properties can be well interpreted as follows: when the pH was 7.4 (more than 4.8 of pK_PAA_), the strong repulsion among -COO^−^ caused swelling of the PAA chains because of its ionization. In contrast, at pH 2.0 (less than 4.8), PAA chains interacted with each other through hydrogen bonding, resulting in volume shrinkage and poor solubility [40].

To explore the dispersion state of NA doped in the PAA network during swelling, the emission fluorescence spectra of solid and filtrate of P(NA-AA)-x-y swelling at different times were investigated. Figure S9 showed the photoluminescence spectra of P(NA-AA)-x-y as a function of swelling time under pH 7.4. Compared with the sample (Figure 1B-b, -d, and -h) without swelling, the swollen solids (Figure S9A, C and E) presented a peak, indicating uniform dispersion of NA with one aggregation state in the PAA frameworks, in which the emission peak positions of the swollen P(NA-AA)−0−0.1, P(NA-AA)−0−1, and P(NA-AA)−0−10 at pH 7.4 were 380.0, 395.0, and 404.4 nm, respectively, implying the tendency of gradual aggregation with an increase in NA-doped amount. Meanwhile, the fluorescent intensities of the swollen solids presented declining trends along with increases in NA-doped amount due to fluorescence quenching caused by its aggregation [17]. On the other hand, the fluorescent intensity of the filtrated solutions showed a slightly increasing trend with an increase in NA-doped amount, indicating a possibility of NA leaching from P(NA-AA) occurring with an increase in doping amount [27]. As shown in Figure S9B, D, and F, similar results can be also observed in other samples P(NA-AA)−7−0.1, P(NA-AA)−7−1, and P(NA-AA)−7−10. In particular, the emission wavelength of P(NA-AA)−7−10 (Figure S9F) solid was much longer than that of filtrate, indicating more aggregations of NA clusters in solid.

Figure S10 showed the photoluminescence spectra of P(NA-AA)-x-y as a function of the swelling time under pH 2.0. As can be seen in Figure S10A, C, and E, the emission spectra of the swollen samples were almost the same as the profiles in Figure S9A, C, and E, indicating the relatively uniform aggregation state of the doped NA in the swollen samples at pH 2.0. The emission peak positions of P(NA-AA)−0−0.1, P(NA-AA)−0−1, and P(NA-AA)−0−10 after shrinking at pH 2.0 were 383.0, 401.0, and 407.0 nm, respectively. Compared with the sample swollen at pH 7.4 (Figure S9A, C, and E), the emission peak position of the solid sample shrunken at pH 2.0 was more redshifted, meaning that NA clusters were more aggregative at pH 2.0 than at pH 7.4. The fluorescent intensity of the shrunken solid at pH 2.0 was weaker than that at pH 7.4 overall, while the fluorescent intensity of the liquid being just the opposite, indicating that more NA molecules fell from the filtrated solid into the liquid at pH 2.0. The reason for these results may be that the PAA network was in a shrunken state owing to the hydrogen bond between carboxyl groups and protons at pH 2.0, easily resulting in extrusion of NA from the PAA network. Similarly, these results can be observed in P(NA-AA)−7−0.1, P(NA-AA)−7−1, and P(NA-AA)−7−10 samples, as shown in Figure S10B, D, and F.

In order to further explore the stability of NA in the swelling process of the PAA network under extreme pH conditions, the emission fluorescent spectra of P(NA-AA)−0−0.1 swelling solid and filtrate with different time were investigated at strong acid (pH 1.0) and alkaline (pH 10.0). As shown in Figure S11, the fluorescent intensity of the solid became gradually weaker with extension of swelling/shrinking time (up to 5 days), while that of the filtrate became stronger, indicating occurrences of the doped NA leaching from P(AA-NA). However, the strong fluorescent intensity of the P(AA-NA) matrix provides a possibility for its potential clinical applications.

**Figure 6:**
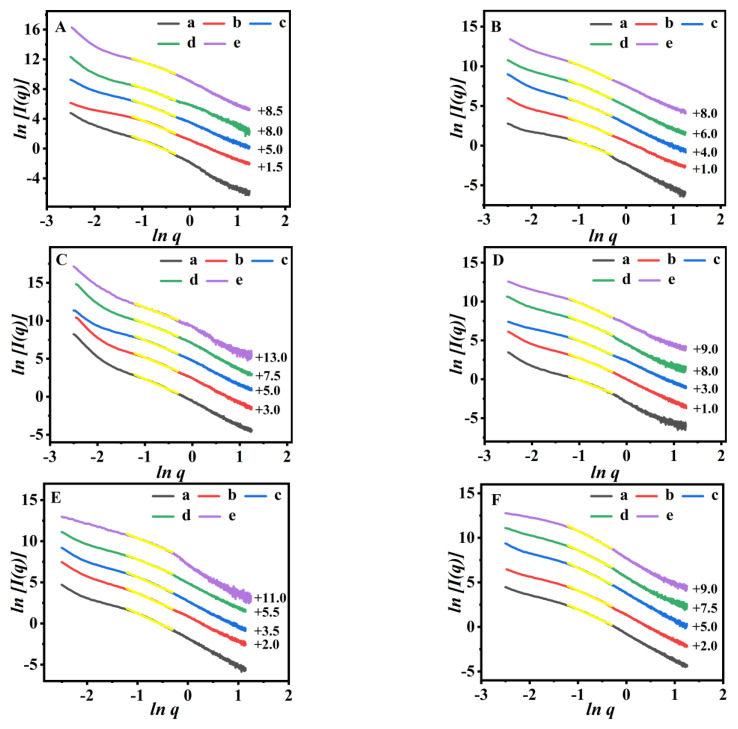
SAXS patterns (yellow lines were fitting curves based on the power low) of the swollen/shrunken (**A**,**B**) P(NA-AA)−0−0.1, (**C**,**D**) P(NA-AA)−0−1, and (**E**,**F**) P(NA-AA)−0−10 under pH 7.4 (**A**,**C,** and **E**) and 2.0 (**B**,**D,** and **F**) with the extended time. (a) 1 h, (b) 3 h, (c) 5 h, (d) 8 h, and (e) 24 h. The vertical offset values were presented in the right *y*-axis.

The SAXS method is a powerful tool to study fractal structures of copolymers [41,42]; therefore, we tried to use SAXS patterns to investigate the impact of pH and swelling/shrinking time on the microstructures of P(NA-AA) matrixes. The fractal evolutions of P(NA-AA)−0−y (y = 0.1, 1, and 10, respectively) in the swelling/shrinking process are shown in Figure 6. The swelling P(NA-AA)−0−y sample showed good mass fractal characteristics in the high-*q* regions.

As shown in Figure 6A, the *D_m_* values of P(NA-AA)−0−0.1 at pH 7.4 decreased gradually with sustained swelling time, showing 2.66, 2.53, 2.43, 2.32, and 2.28 for 1, 3, 5, 8, and 24 h, respectively. These findings implied that the networks of P(NA-AA)−0−0.1 became loose structures [43], consistent with the demonstrations of the SEM images (Figure 3A). Similar phenomena were also observed in other samples when the swelling time was prolonged from 1 to 24 h: the *D_m_* value decreased from 2.70 to 2.36 for P(NA-AA)−0−1 (Figure 6C) and from 2.84 to 2.45 for P(NA-AA)−0−10 (Figure 6E), while, as shown in Figure 6B, the *D_m_* values of P(NA-AA)−0−0.1 at pH 2.0 were 2.29, 2.38, 2.48, 2.51, and 2.54 for 1, 3, 5, 8, and 24 h, respectively, suggesting their transformations from loose structures to dense networks [32], consistent with the results of SEM (Figure 3B). When the shrinking time was prolonged from 1 to 24 h, the *D_m_* value increased from 2.42 to 2.61 for P(NA-AA)−0−1 (Figure 6D) and from 2.47 to 2.79 for P(NA-AA)−0−10 (Figure 6F). A similar phenomenon was also observed in P(NA-AA)−1−0.1 (Figure S12A,B) and P(NA-AA)−7−0.1(Figure S12C,D). Obviously, the main reason for the mentioned observations is that, at a high pH, PAA was in highly swollen state due to the electrostatic repulsions between the ionized carboxylic acid. On the contrary, at a low pH, the network of PAA presented shrinkage due to hydrogen bonding between -COOH and proton.

As Kang reports [44,45], the dispersion state of CaCO_3_ in polypropylene composites was quantitatively evaluated by fractal analysis; therefore, we also tried to use the fractal value to describe the dispersion state of NA doped in PAA. As shown in Figure 6, the *D_m_* value of the P(NA-AA) swollen at 1 h increased from 2.66 for P(NA-AA)−0−0.1 (Figure 6A-a) to 2.70 for P(NA-AA)−0−1 (Figure 6C-a) and continually increased to 2.84 for P(NA-AA)−0−10 (Figure 6E-a), suggesting that the aggregated NA clusters became dense structures. Similar results also appeared at swelling times of 3, 5, 8, and 24 h, as shown in Figure 6 and Figure S12.

**Figure 7:**
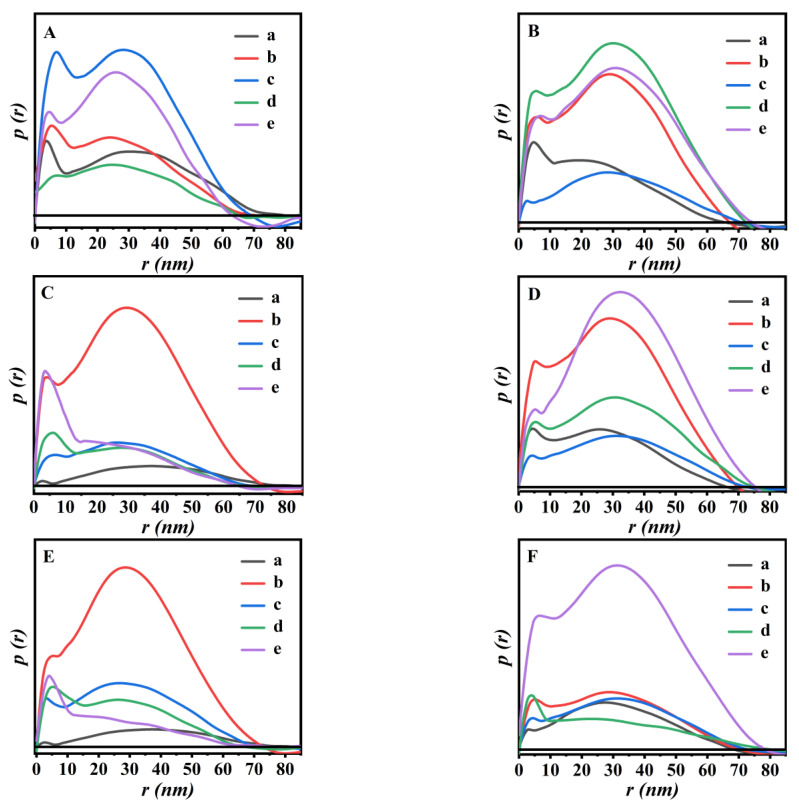
PDDF profiles of the swollen/shrunken P(NA-AA)−0−0.1 (**A**,**D**), P(NA-AA)−0−1(**B**,**E**), and P(NA-AA)−0−10 (**C**,**F**) under pH 7.4 (**A**,**B,** and **C**) and 2.0 (**D**,**E,** and **F**) with the extended time. (a) 1 h, (b) 3 h, (c) 5 h, (d) 8 h, and (e) 24 h.

In addition, the PDDF profiles of P(NA-AA) swelling/shrinking at different times were shown in Figure 7. The PDDF curves of the obtained P(NA-AA) presented two partially resolved peaks, indicating that it was composed of two units: the former peak (*r* ≈ 4 nm) represented NA cluster and the latter (*r* ≈ 26 nm) represented PAA.

As profiled in Figure 7A, the *R_g_* value of the cross-linked networks of P(NA-AA)−0−0.1 swelling at pH 7.4 for 1 h was around 24.01 nm but decreased to 17.32 nm with extension of swelling time of up to 24 h. This variable trend is consistent with Cheng’s demonstration [32], indicating that the size of the aggregates formed *at the initial swelling stage* of P(NA-AA)−0−0.1 was large. The main reason is that the PAA network extended to a straight chain structure with an increase in the swelling time until the swelling equilibrium. As seen in Figure 7B, the *R_g_* value of the cross-linked networks of P(NA-AA)−0−0.1 shrinking at pH 2.0 for 1 h was around 18.26 nm but increased to 22.95 nm with extension of shrinking time of up to 24 h, indicating that the size of the aggregates formed *in the initial shrinkage stage* of P(NA-AA)−0−0.1 was small due to folding of the P(NA-AA) network with an increase in shrinkage time. A similar phenomenon was also observed in P(NA-AA)−0−1 (Figure 7C,D), P(NA-AA)−0−10 (Figure 7E,F), P(NA-AA)−1−0.1 (Figure S13A,B), and P(NA-AA)−7−0.1 (Figure S13C,D).

As shown in Figures S14 and S15, Kratky plots might be used to further illustrate these findings. As shown in Figure S14A–E, a plateau presented at *q* > 0.8 nm^−1^ for the swollen P(NA-AA) at pH 7.4 and then monotonically increased in the range of high *q*-range; their intensities increased gradually in larger *q*-range with extension of swelling time, identifying occurrences of unfolding network in the swollen P(NA-AA) [46]. Conversely, the shrunken P(NA-AA) at pH 2.0 (Figure S15) retained its Gaussian-like shape at low *q*-ranges and exhibited a well-defined maximum in the high *q*-regions, indicating the appearance of aggregates or folds structures [47,48].

### 3.4. In Vitro Drug-Releasing Performances

The IBU loading capacities of P(NA-AA)−0−0.1, P(NA-AA)−0−1, P(NA-AA)−0−10, P(NA-AA)−1−0.1, P(NA-AA)−1−1, P(NA-AA)−1−1P(NA-AA)−1−10, P(NA-AA)−7−0.1, P(NA-AA)−7−1, and P(NA-AA)−7−10 were 5.93, 5.47, 5.66, 5.31, 4.38, 5.51, 5.17, and 4.02%, respectively, showing a low drug loading capacity, which is mainly due to the low specific surface area (around 118.1 m^2^/g) of PAA and weak interactions (physical adsorption and hydrogen bonding) between IBU and PAA [49], resulting in unfavorable drug delivery through the used polymer.

**Figure 8:**
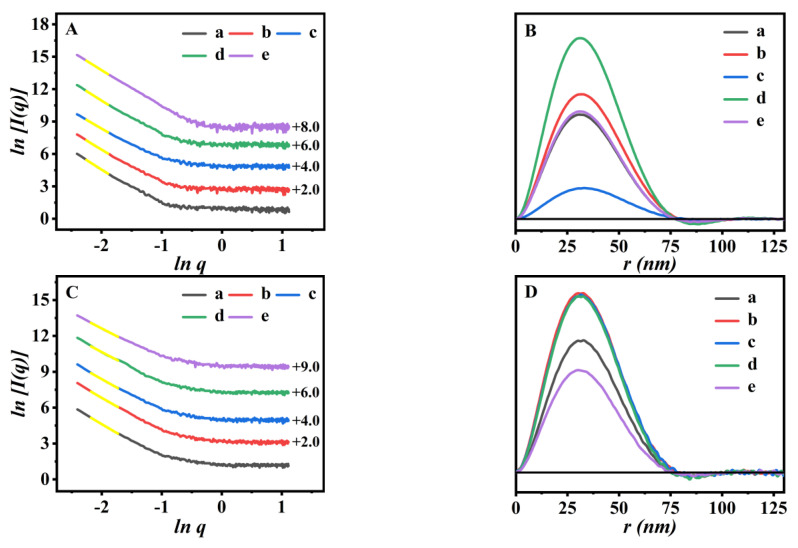
SAXS patterns (yellow lines were fitting curves based on the power low) (**A**) and their PDDF profiles of the drug-loaded samples (**B**). (a) I/P(NA-AA)−0−0.1, (b) I/P(NA-AA)−0−1, (c) I/P(NA-AA)−0−10, (d) I/P(NA-AA)−1−0.1, and (e) I/P(NA-AA)−7−0.1. SAXS patterns (yellow lines are fitting curves) (**C**) of I/P(NA-AA)−0−0.1 in PBS solution (pH 2.0) with drug release time intervals, respectively, and their PDDF profiles (**D**). (a) 1h, (b) 3 h, (c) 5 h, (d) 8 h, and (e) 24 h.

The microstructures of the drug-loaded sample undergo fractal evolution accordingly. Therefore, the fractal analysis and PDDF curve are introduced to further describe the drug-loaded samples. Figure 8A displayed that the *Ds* values of I/P(NA-AA)−0−0.1, I/P(NA-AA)−0−1, I/P(NA-AA)−0−10, I/P(NA-AA)−1−0.1, and I/P(NA-AA)−7−0.1 were 2.46, 2.61, 2.77, 2.49, and 2.53, respectively. These results indicated that their fractal characteristics varied from mass fractal of the pre-IBU-loaded sample (seen in Figure 4A) to surface fractal, suggesting the transformation of the crosslinked structures from high porosity to densely rough surface. One of the possible reasons may be due to the successful IBU-loading into the cross-linked networks of the P(NA-AA)-x-y [50,51].

In addition, the detailed morphological information of P(NA-AA) (including the shape and size of the cross-linked network) could be speculated based on the PDDF profiles. As shown in Figure 8B, the bell-shaped peak of the IBU-loaded sample remained ellipsoid, almost identical to that of the unloaded samples (as shown in Figure 4B,D). Meanwhile, the *R_g_* size of the aggregated networks in the IBU-loaded samples increased to 26.58, 27.14, 27.99, 26.71, and 26.87 nm for I/P(NA-AA)−0−0.1, I/P(NA-AA)−0−1, I/P(NA-AA)−0−10, I/P(NA-AA)−1−0.1, and I/P(NA-AA)−7−0.1, respectively, larger than for pure samples.

Based on the drug release profiles, the fractal and PDDF curves were also used to further describe the drug-releasing performances of I/P(NA-AA)−0−0.1 at pH 2.0. According to the fitting curve, the correlation of low-*q* regions was good, and all samples presented the typical mass fractal characteristics. As shown in Figure 8C, the *D_m_* values of P(NA-AA)−0−0.1 at pH 2.0 were about 2.94 at a release time of 1 h, 2.87 at 3 h, 2.81 at 5 h, 2.62 at 8 h, and 2.58 at 24 h, respectively, indicating that the *D_m_* values fell progressively with sustained IBU-release. These results revealed the transition from a rough surface to loosely ambiguous interfaces, consistent with our earlier research [52]. Figure 8D demonstrates that the PDDF curves for I/P(NA-AA)−0−0.1 had symmetrical profiles during the sustained drug-releasing process, showing an ellipsoidal shape [29] identical to that of the samples before drug loading (as shown in Figure 8B-a). Meanwhile, the *R_g_* values ranged from 26.46 (0 h) to 26.28 nm (24 h), attributed to their shrinkage states at acidic medium.

**Figure 9:**
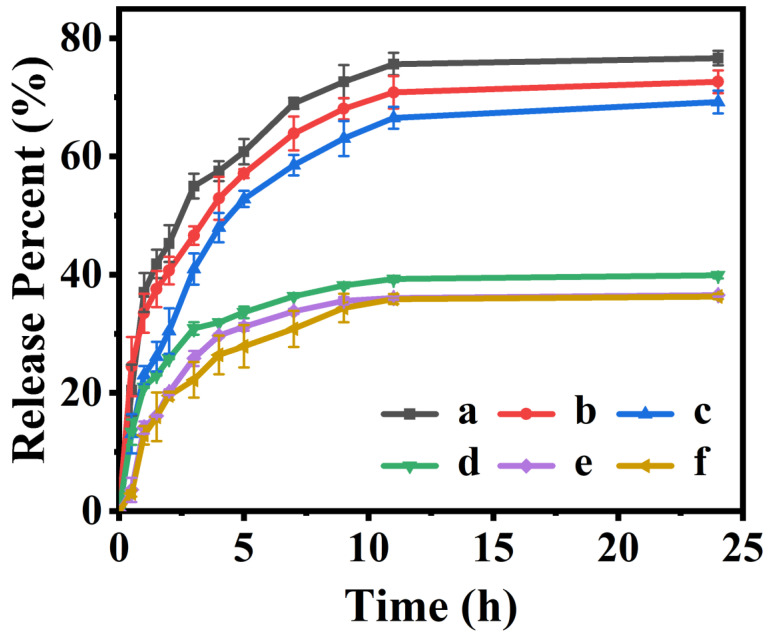
Cumulative IBU-releasing profiles from drug-loaded P(NA-AA)−0−y at 37.0 °C under pH 2.0 (y = 0.1 (a), 1.0 (b), and 10.0 (c)) and 7.4 (y = 0.1 (d), 1.0 (e), and 10.0 (f)).

As illustrated in Figure 9, the in vitro drug release behaviors of drug-loaded P(NA-AA) were investigated in PBS with pH values of 7.4 and 2.0 at 37 °C. A significant controlled-release performance via pH-stimulation of all samples can be observed in which the IBU-releasing equilibrium amount from P(NA-AA) at pH 7.4 was much higher than that at pH 2.0. In detail, the cumulative IBU-released amounts of P(NA-AA)−0−0.1, P(NA-AA)−0−1, and P(NA-AA)−0−10 at pH 7.4 were about 39.9, 36.5, and 36.3%, respectively, but increased to 76.6, 72.6, and 69.2% at pH 2.0. Similar results were also observed in Figure S16A,B. The performances of the ionization and deionization of the carboxyl groups of the P(NA-AA) might interpret these pH-sensitive release features, which are mostly dependent on the unique swelling–shrinking behavior under different pH solutions. As discussed above, under alkaline conditions, the carboxyl groups are ionized and electrostatic repulsion forces the network to expand, resulting in IBU-loading being released from the crosslinked network, predominantly by diffusion, while the carboxyl group is protonated under acidic conditions and the cross-linked network is in a state of shrinking, which leads to effective extrusion and release of IBU.

As shown in Figures S17 and S18, the emission spectra of the drug-released P(NA-AA) were almost the same as those in Figures S9 and S10. A peak of P(NA-AA) appeared after drug-release, indicating uniform dispersion of NA with one aggregation state in the PAA frameworks, in which the emission peak positions of the swollen P(NA-AA)−0−0.1, P(NA-AA)−0−1, and P(NA-AA)−0−10 at pH 7.4 were 381.7, 398.1, and 415.0 nm, but, at pH 2.0, they were 382.5, 398.5, and 416.0 nm, respectively, meaning that NA clusters were more aggregative at pH 2.0 than at pH 7.4.

On the other hand, the fluorescence intensity of the solid at pH 2.0 was weaker than that of the solid at pH 7.4 overall, while the fluorescent intensity of the liquid was just the opposite, indicating that more NA fell from the filtrated solid into the liquid at pH 2.0. The reason may be related to the aggregated state of the PAA networks, with occurrences of the hydrogen bonds between carboxyl groups and protons at pH 2.0 resulting in NA-leaching from the networks. Similarly, these results can be observed in P(NA-AA)−7−0.1, P(NA-AA)−7−1, and P(NA-AA)−7−10, as shown in Figures S17 and S18.

### 3.5. Cytotoxicity Properties

**Figure 10:**
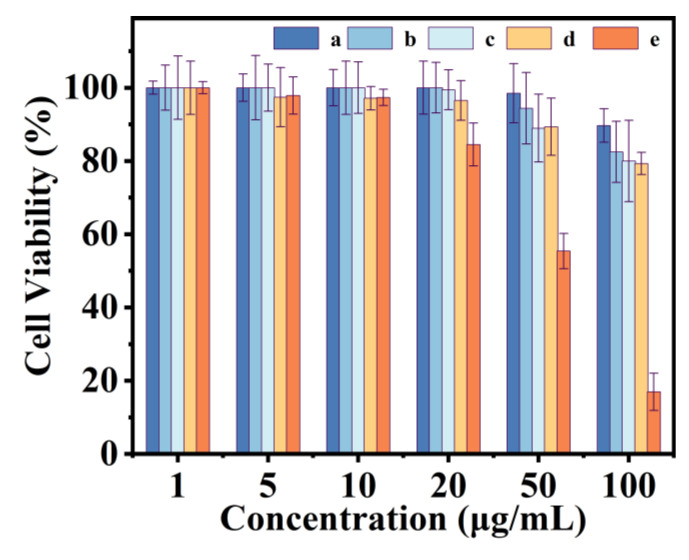
Cytotoxicity of synthetic materials against Hela cells after 48 h treatment. (a) PAA, (b) P(NA-AA)−0−0.1, (c) P(NA-AA)−0−1, (d) P(NA-AA)−0−10, and (e) NA.

Considering that low toxicity of an ideal drug carrier should be requested for cell imaging and moving to in vivo experiments, a CCK-8 assay via HeLa cells was conducted to evaluate the cytotoxicity of the carrier. Figure 10 displays the cell viabilities after exposing HeLa cells to increasing concentrations (1–100 µg/mL) of carrier for 48 h. The cell viability of PAA (Figure 10-a) remained 90% at concentrations as high as 100 µg/mL, indicating minimal cytotoxicity of PAA, consistent with previously reported findings [53]. A dose-dependent decrease pattern in HeLa cell viability was observed for NA molecules. The cell viability was more than 85% with concentrations of up to 20 µg/mL, although an increase in cell toxicity was observed for more concentrated; thus, the very low amount was doped to the study sample P(NA-AA). Since the toxicity of the P(NA-AA) increased further to Hela cells due to the NA doping amount being enhanced from 0.1 to 10 wt%, P(NA-AA)−0−0.1 (Figure 10b), -1 (Figure 10c), and -10 (Figure 10d) displayed 82 %, 80%, and 79% cell viability after 48 h even at 100 µg/mL concentration. Accordingly, these cytotoxicity results indicate that they have very low cytotoxicity, which contributes to potential in vivo applications.

### 3.6. In Vitro Cellular Uptake Behaviors

**Figure 11:**
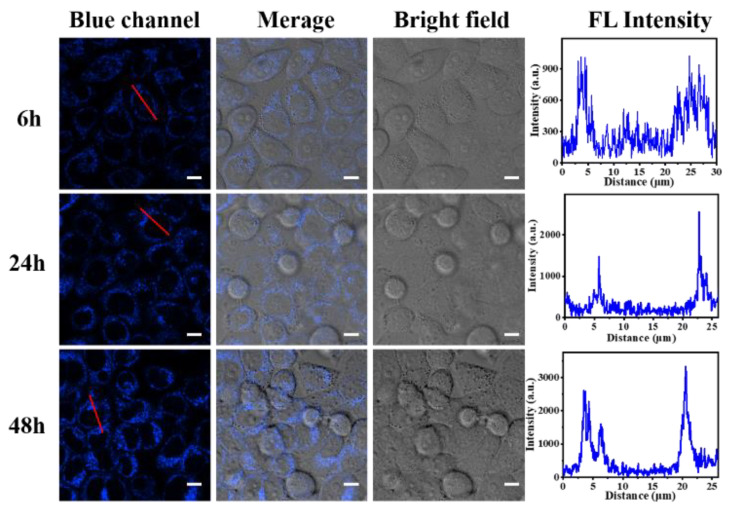
Time-dependent CLSM images of HeLa cells incubated with P(NA-AA)−0−0.1 at a concentration of 20 µg/mL. The scale bars are 10 µm.

Based on the above cytotoxicity results, a concentration of 20 µg/mL was considered to be extremely minimally toxic and safe; hence, uptake of P(NA-AA)−0−0.1 by HeLa cells as a function of time was investigated at this concentration. As shown in Figure 11, after incubation with P(NA-AA)−0−0.1, the blue fluorescence signal was observed in the cytoplasm of HeLa cells by 405 nm excitation. Compared with the control group (Figure S19), the fluorescent intensity of P(NA-AA)−0−0.1 after incubation significantly increased with prolonged time, indicating that P(NA-AA)−0−0.1 could be internalized in a time-dependent manner. These results suggest that P(NA-AA)−0−0.1 is a good carrier for living cell imaging and has potential for in vivo imaging.

## 4. Conclusions

Fluorescent tracking functionalization of PAA was carried out via a doping method, and the dispersion behaviors of the doped NA in the PAA frameworks were elucidated via their fractal evolution combined with other characterizations. The results showed that the P(NA-AA) matrix presented a cross-linking network structure, while the emission peak position red-shifted from 380.0 to 409.2 nm when the NA-doped amount increased from 0.1 to 10%, accompanied by an increase in *D_m_* values from 2.25 to 2.65 and fluorescence lifetime from 2.102 ns to 2.389 (and 11.350 ns). These demonstrations suggest that the NA clusters dispersed in the PAA frameworks were more aggregative with an increase in doped NA amount. Meanwhile, the *SR* values of the obtained P(NA-AA) varied from 293 to 4472% (up to swelling equilibrium) at pH 7.4, along with decreases in the *D_m_* values from 2.66 to 2.28. These phenomena indicated that the resultant P(NA-AA) had an excellent pH-responsive property, but their aggregative networks became gradually looser with extension of swelling time. The IBU-releasing behavior indicated that the cumulative release at pH 2.0 was significantly higher than that at pH 7.4. Their stable fluorescent intensity during drug-loading and releasing achieved a good labeling effect. Especially, the fractal evolutions of IBU-loaded P(NA-AA) during the release process presented transformation behaviors from *D_s_* to *D_m_*. CCK-8 assay suggested that the preparation of fluorescent polymer had a low-toxicity. Cell uptake study showed that P(NA-AA) could be internalized into the cytoplasm in a time-dependent manner. Hence, these pH-responsive fluorescent polymers could be expected to be applied as a potential drug carrier in biomedical fields.

## Data Availability

The data presented during the study are available on request from the corresponding author.

## Supplementary Materials

The following supporting information can be downloaded at: https://www.mdpi.com/article/10.3390/polym15030596/s1, including standard calibration curves of drug loading and releasing, FT-IR, TG, PL, SAXS characterizations, summary of drug loading rate and cumulative release rate, and time-dependent CLSM images of HeLa cells of P(NA-AA) associated with this article. Figure S1: HPLC standard curve of IBU concentration and corresponding Regression equations (insert). Figure S2: HPLC.; Figure S2: The standard curves of IBU concentration in PBS at pH 7.4 (A) and pH 2.0 (B), corresponding their Regression equations (insert). Figure S3: Emission spectra of NA in acetonitrile solution at different concentrations (A), (a) ACN, (b) 1.0×10^−4^ M, (c) 2.5×10^−4^ M, (d) 3.3×10^−3^ M, (e) 5.0×10^−3^ M, (f) 8.3×10^−3^ M, (g) 1.0×10^−2^ M, (h) 5.0×10^−2^ M, (i) 1.0×10^−1^ M, (j) NA solid. The relationships between their concentration values and wavelength values (B). Figure S4: Emission spectra of P(NA-AA)−1−y (A) and P(NA-AA)−7−y (B) with various doped amount of NA, (a) P(NA-AA)-x-0.05, (b) P(NA-AA)-x-0.1, (c) P(NA-AA)-x-0.5, (d) P(NA-AA)-x-1, (e) P(NA-AA)-x-3, (f) P(NA-AA)-x-5, (g) P(NA-AA)-x-7, and (h) P(NA-AA)-x-10. The emission spectra of the NA-PAAs mixed by physical method (C), (a) NA-PAA-0.05, (b) NA-PAA-0.1, (c) NA-PAA-0.5, (d) NA-PAA-1, (e) NA-PAA-3, (f) NA-PAA-5, (g) NA-PAA-7, and (h) NA-PAA-10. The relationships between the wavelength values of the characteristic peak and the doped-NA amount in PAA (D), (a) P(NA-AA)−0−y, (b) P(NA-AA)−1−y, (c) P(NA-AA)−7−y, and (d) NA-PAA-y. Figure S5: Kratky plots(A) of (a) PAA, (b) P(NA-AA)−0−0.1, (c) P(NA-AA)−0−1, (d) P(NA-AA)-0-7, and (e) P(NA-AA)−0−10. Kratky plots (A) of (a) P(NA-AA)−0−0.1, (b) P(NA-AA)−1−0.1, (c) P(NA-AA)-3-0.1, and (d) P(NA-AA)−7−0.1. Figure S6: TG (A) and DTG (B) plots of (a) PAA, (b) P(NA-AA)−0−0.1, (c) P(NA-AA)−0−1, (d) P(NA-AA)−0−10 and (e) NA. Figure S7: FT-IR spectra of (a) PAA, (b) P(NA-AA)−0−0.1, (c) P(NA-AA)−0−1, (d) P(NA-AA)−0−10, and (e) NA. Figure S8: SR profiles of P(NA-AA)−1−y (A) and P(NA-AA)−7−y (B) at 37.0 °C under pH 7.4 (y = 0.1 (a), 1.0 (b), 10.0 (c)) and 2.0 (y = 0.1 (d), 1.0 (e), and 10.0 (f)). Figure S9: PL spectra of (A) P(NA-AA)−0−0.1, (B) P(NA-AA)−7−0.1, (C) P(NA-AA)−0−1, (D) P(NA-AA)−7−1, (E) P(NA-AA)−0−10, and (F) P(NA-AA)−7−10 with different swelling time under pH 7.4. Notes: S- represents the swollen solid and L- the filtrated solution obtained from the swelling system over time. Figure S10: PL spectra of (A) P(NA-AA)−0−0.1, (B) P(NA-AA)−7−0.1, (C) P(NA-AA)−0−1, (D) P(NA-AA)−7−1, (E) P(NA-AA)−0−10, and (F) P(NA-AA)−7−10 with different shrinking time under pH 2.0. Figure S11: PL spectra of P(NA-AA)−0−0.1 with different swelling/shrinking time under pH 10.0 (A) and pH 1.0 (B). Figure S12: SAXS patterns (yellow lines were fitting curves based on the power low) of the swollen/shrunken (A and B) P(NA-AA)−1−0.1, and (C and D) P(NA-AA)−7−0.1 under pH 7.4 (A, and C) and 2.0 (B, and D) with the extended time. (a) 1 h, (b) 3 h, (c) 5 h, (d) 8 h, and (e) 24 h. The vertical offset values were presented in the Y-axis. Figure S13: PDDF profiles of the swollen/shrunken P(NA-AA)−1−0.1 (A and C), and P(NA-AA)−7−0.1 (B and D) under pH 7.4 (A, and B) and 2.0 (D, and E) with the extended time. (a) 1 h, (b) 3 h, (c) 5 h, (d) 8 h, and (e) 24 h. Figure S14: Kratky plots of the swollen P(NA-AA)−0−0.1 (A), P(NA-AA)−0−1 (B), P(NA-AA)−0−10 (C), P(NA-AA)−1−0.1 (D), and P(NA-AA)−7−0.1 (E) under pH 7.4 with the extension of the swelling time. (a) 1 h, (b) 3 h, (c) 5 h, (d) 8 h, and (e) 24 h. Figure S15: Kratky plots of the shrunken P(NA-AA)−0−0.1(A), P(NA-AA)−0−1 (B), P(NA-AA)−0−10 (C), P(NA-AA)−1−0.1 (D), and P(NA-AA)−7−0.1 (E) under pH 2.0 with the extension of the shrinking time. (a) 1 h, (b) 3 h, (c) 5 h, (d) 8 h, and (e) 24 h. Figure S16: Cumulative IBU-releasing profiles from drug-loaded P(NA-AA)−1−y (A) and P(NA-AA)−7−y (b) at 37.0 °C under pH 2.0 (y = 0.1 (a), 1.0 (b), 10.0 (c)) and 7.4 (y = 0.1 (d), 1.0 (e), and 10.0 (f)). Figure S17: PL spectra of the drug-released (A) P(NA-AA)−0−0.1, (B) P(NA-AA)−7−0.1, (C) P(NA-AA)−0−1, (D) P(NA-AA)−7−1, (E) P(NA-AA)−0−10, and (F) P(NA-AA)−7−10 with different releasing time under pH 7.4. Notes: As described in experimental methods, the drug loaded samples were immersed in PBS system, 1.0 mL of releasing solution was withdrawn at a predetermined time. After filtered, the obtained solid was defined S, and corresponding filtrate liquid was defined L. Figure S18: PL spectra of the drug-released (A) P(NA-AA)−0−0.1, (B) P(NA-AA)−7−0.1, (C) P(NA-AA)−0−1, (D) P(NA-AA)−7−1, (E) P(NA-AA)−0−10, and (F) P(NA-AA)−7−10 with different releasing time under pH 2.0. Figure S19: Time-dependent CLSM images of HeLa cells incubated as controlled samples. The scale bars are 10 µm. Table S1. Summary of P(NA-AA)-x-y drug loading rate and cumulative release rate under pH 7.4 and pH 2.0.
