# Supplementary material for: Dispersion Performances and Fluorescent Behaviors of Naphthalic Anhydride Doped in Poly(acrylic acid) Frameworks for pH-Sensitive Ibuprofen Delivery via Fractal Evolution
Source: Polymers (Basel). 2023 Jan 24;15(3):596. doi: 10.3390/polym15030596 (PMC9921450; doi:10.3390/polym15030596)
Supplement: Supplementary file 1 [file polymers-15-00596-s001.zip › polymers-2096356-supplementary.pdf]

Electronic Supplementary Information

Figure S1: HPLC standard curve of IBU concentration and corresponding Regression equations (insert).

Figure S2: HPLC.; Figure S2: The standard curves of IBU concentration in PBS at pH 7.4 (A) and pH 2.0 (B), corresponding their Regression equations (insert).

Figure S3: Emission spectra of NA in acetonitrile solution at different concentrations (A), (a) ACN, (b) 1.0×10^{-4} M, (c) 2.5×10^{-4} M, (d) 3.3×10^{-3} M, (e) 5.0×10^{-3} M, (f) 8.3×10^{-3} M, (g) 1.0×10^{-2} M, (h) 5.0×10^{-2} M, (i) 1.0×10^{-1} M, (j) NA solid. The relationships between their concentration values and wavelength values (B).

Figure S4: Emission spectra of P(NA-AA)-1-y (A) and P(NA-AA)-7-y (B) with various doped amount of NA, (a) P(NA-AA)-x-0.05, (b) P(NA-AA)-x-0.1, (c) P(NA-AA)-x-0.5, (d) P(NA-AA)-x-1, (e) P(NA-AA)-x-3, (f) P(NA-AA)-x-5, (g) P(NA-AA)-x-7, and (h) P(NA-AA)-x-10. The emission spectra of the NA-PAAs mixed by physical method (C), (a) NA-PAA-0.05, (b) NA-PAA-0.1, (c) NA-PAA-0.5, (d) NA-PAA-1, (e) NA-PAA-3, (f) NA-PAA-5, (g) NA-PAA-7, and (h) NA-PAA-10. The relationships between the wavelength values of the characteristic peak and the doped-NA amount in PAA (D), (a) P(NA-AA)-0-y, (b) P(NA-AA)-1-y, (c) P(NA-AA)-7-y, and (d) NA-PAA-y.

Figure S5: Kratky plots(A) of (a) PAA, (b) P(NA-AA)-0-0.1, (c) P(NA-AA)-0-1, (d) P(NA-AA)-0-7, and (e) P(NA-AA)-0-10. Kratky plots (A) of (a) P(NA-AA)-0-0.1, (b) P(NA-AA)-1-0.1, (c) P(NA-AA)-3-0.1, and (d) P(NA-AA)-7-0.1.

Figure S6: TG (A) and DTG (B) plots of (a) PAA, (b) P(NA-AA)-0-0.1, (c) P(NA-AA)-0-1, (d) P(NA-AA)-0-10 and (e) NA.

Figure S7: FT-IR spectra of (a) PAA, (b) P(NA-AA)-0-0.1, (c) P(NA-AA)-0-1, (d) P(NA-AA)-0-10, and (e) NA.

Figure S8: SR profiles of P(NA-AA)-1-y (A) and P(NA-AA)-7-y (B) at 37.0 °C under pH 7.4 (y = 0.1 (a), 1.0 (b), 10.0 (c)) and 2.0 (y = 0.1 (d), 1.0 (e), and 10.0 (f)).

Figure S9: PL spectra of (A) P(NA-AA)-0-0.1, (B) P(NA-AA)-7-0.1, (C) P(NA-AA)-0-1, (D) P(NA-AA)-7-1, (E) P(NA-AA)-0-10, and (F) P(NA-AA)-7-10 with different swelling time under pH 7.4. Notes: S- represents the swollen solid and L- the filtrated solution obtained from the swelling system over time.

Figure S10: PL spectra of (A) P(NA-AA)-0-0.1, (B) P(NA-AA)-7-0.1, (C) P(NA-AA)-0-1, (D) P(NA-AA)-7-1, (E) P(NA-AA)-0-10, and (F) P(NA-AA)-7-10 with different shrinking time under pH 2.0.

Figure S11: PL spectra of P(NA-AA)-0-0.1 with different swelling/shrinking time under pH 10.0 (A) and pH 1.0 (B).

Figure S12: SAXS patterns (yellow lines were fitting curves based on the power low) of the swollen/shrunken (A and B) P(NA-AA)-1-0.1, and (C and D) P(NA-AA)-7-0.1 under pH 7.4 (A, and C) and 2.0 (B, and D) with the extended time. (a) 1 h, (b) 3 h, (c) 5 h, (d) 8 h, and (e) 24 h. The vertical offset values were presented in the Y-axis.

Figure S13: PDDF profiles of the swollen/shrunken P(NA-AA)-1-0.1 (A and C), and P(NA-AA)-7-0.1 (B and D) under pH 7.4 (A, and B) and 2.0 (D, and E) with the extended time. (a) 1 h, (b) 3 h, (c) 5 h, (d) 8 h, and (e) 24 h.

Figure S14: Kratky plots of the swollen P(NA-AA)-0-0.1 (A), P(NA-AA)-0-1 (B), P(NA-AA)-0-10 (C), P(NA-AA)-1-0.1 (D), and P(NA-AA)-7-0.1 (E) under pH 7.4 with the extension of the swelling time. (a) 1 h, (b) 3 h, (c) 5 h, (d) 8 h, and (e) 24 h.

Figure S15: Kratky plots of the shrunken P(NA-AA)-0-0.1(A), P(NA-AA)-0-1 (B), P(NA-AA)-0-10 (C), P(NA-AA)-1-0.1 (D), and P(NA-AA)-7-0.1 (E) under pH 2.0 with the extension of the shrinking time. (a) 1 h, (b) 3 h, (c) 5 h, (d) 8 h, and (e) 24 h.

Figure S16: Cumulative IBU-releasing profiles from drug-loaded P(NA-AA)-1-y (A) and P(NA-AA)-7-y (b) at 37.0 °C under pH 2.0 (y = 0.1 (a), 1.0 (b), 10.0 (c)) and 7.4 (y = 0.1 (d), 1.0 (e), and 10.0 (f)).

Figure S17: PL spectra of the drug-released (A) P(NA-AA)-0-0.1, (B) P(NA-AA)-7-0.1, (C) P(NA-AA)-0-1, (D) P(NA-AA)-7-1, (E) P(NA-AA)-0-10, and (F) P(NA-AA)-7-10 with different releasing time under pH 7.4. Notes: As described in experimental methods, the drug loaded samples were immersed in PBS system, 1.0 mL of releasing solution was withdrawn at a predetermined time. After filtered, the obtained solid was defined S, and corresponding filtrate liquid was defined L.

Figure S18: PL spectra of the drug-released (A) P(NA-AA)-0-0.1, (B) P(NA-AA)-7-0.1, (C) P(NA-AA)-0-1, (D) P(NA-AA)-7-1, (E) P(NA-AA)-0-10, and (F) P(NA-AA)-7-10 with different releasing time under pH 2.0.

Figure S19: Time-dependent CLSM images of HeLa cells incubated as controlled samples. The scale bars are 10 μm.

Table S1. Summary of P(NA-AA)-x-y drug loading rate and cumulative release rate under pH 7.4 and pH 2.0.

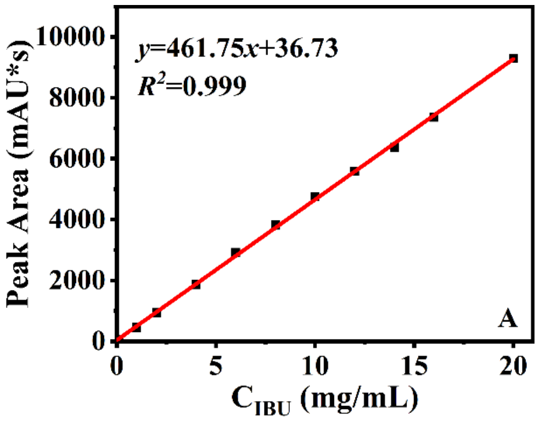

Figure S1. HPLC standard curve of IBU concentration and corresponding Regression

equations (insert).

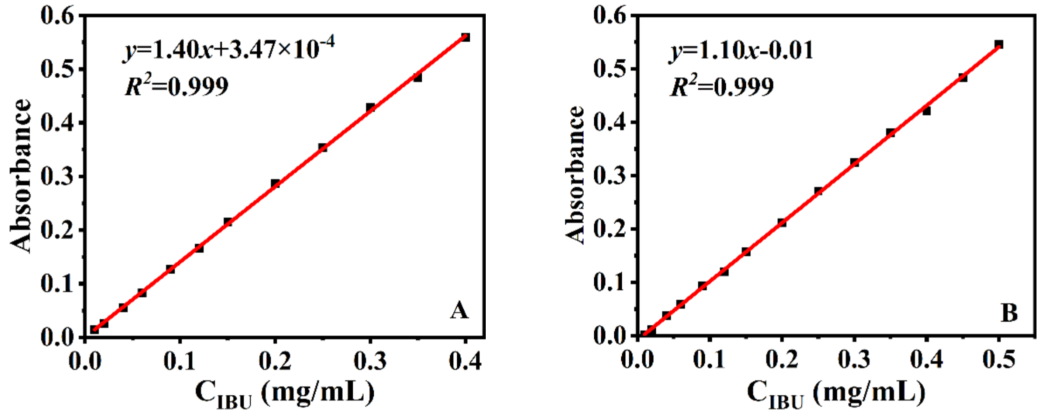

Figure S2. The standard curves of IBU concentration in PBS at pH 7.4 (A) and pH 2.0 (B), corresponding their Regression equations (insert).

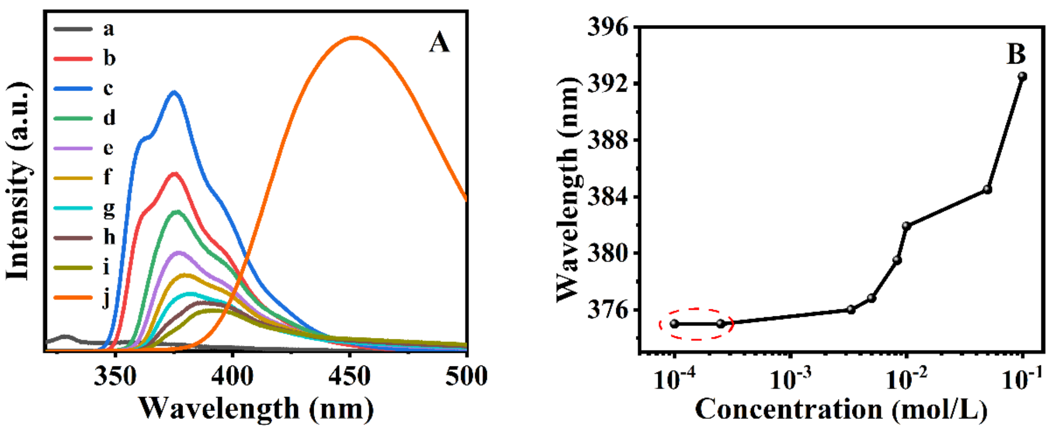

Figure S3. Emission spectra of NA in acetonitrile solution at different concentrations (A), (a) ACN, (b) 1.0×10^{-4} M, (c) 2.5×10^{-4} M, (d) 3.3×10^{-3} M, (e) 5.0×10^{-3} M, (f) 8.3×10^{-3} M, (g) 1.0×10^{-2} M, (h) 5.0×10^{-2} M, (i) 1.0×10^{-1} M, (j) NA solid. The relationships between their concentration values and wavelength values (B).

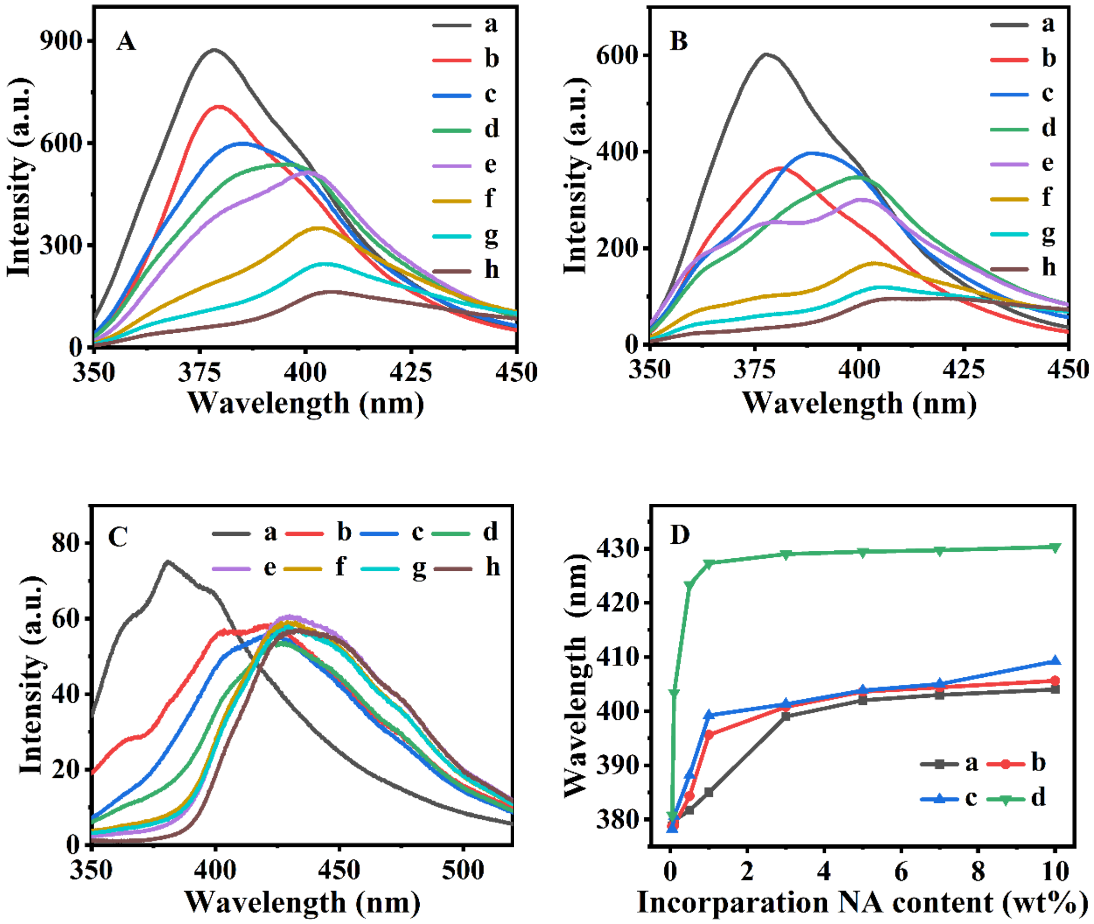

Figure S4. Emission spectra of P(NA-AA)-1-y (A) and P(NA-AA)-7-y (B) with various doped amount of NA, (a) P(NA-AA)-x-0.05, (b) P(NA-AA)-x-0.1, (c) P(NA-AA)-x-0.5, (d) P(NA-AA)-x-1, (e) P(NA-AA)-x-3, (f) P(NA-AA)-x-5, (g) P(NA-AA)-x-7, and (h) P(NA-AA)-x-10. The emission spectra of the NA-PAAs mixed by physical method (C), (a) NA-PAA-0.05, (b) NA-PAA-0.1, (c) NA-PAA-0.5, (d) NA-PAA-1, (e) NA-PAA-3, (f) NA-PAA-5, (g) NA-PAA-7, and (h) NA-PAA-10. The relationships between the wavelength values of the characteristic peak and the doped-NA amount in PAA (D), (a) P(NA-AA)-0-y, (b) P(NA-AA)-1-y, (c) P(NA-AA)-7-y, and (d) NA-PAA-y.

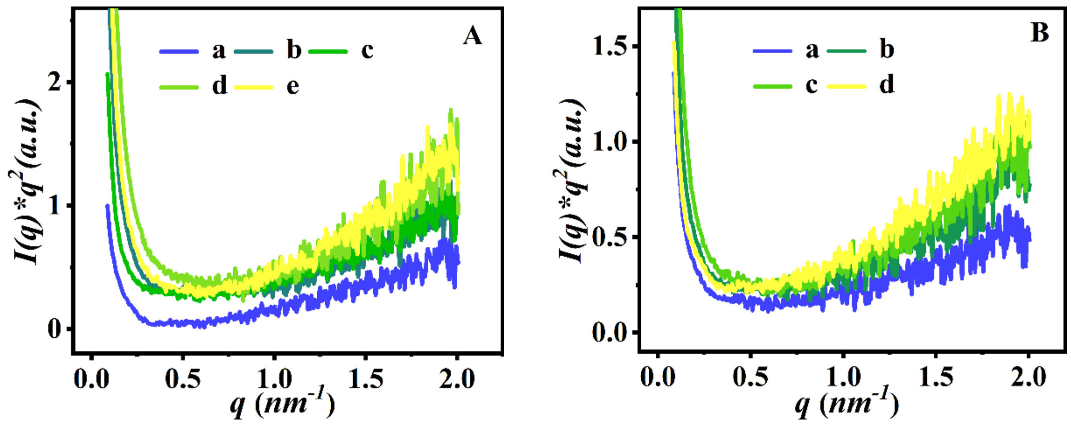

Figure S5. Kratky plots(A) of (a) PAA, (b) P(NA-AA)-0-0.1, (c) P(NA-AA)-0-1, (d) P(NA-AA)-0-7, and (e) P(NA-AA)-0-10. Kratky plots (A) of (a) P(NA-AA)-0-0.1, (b) P(NA-AA)-1-0.1, (c) P(NA-AA)-3-0.1, and (d) P(NA-AA)-7-0.1.

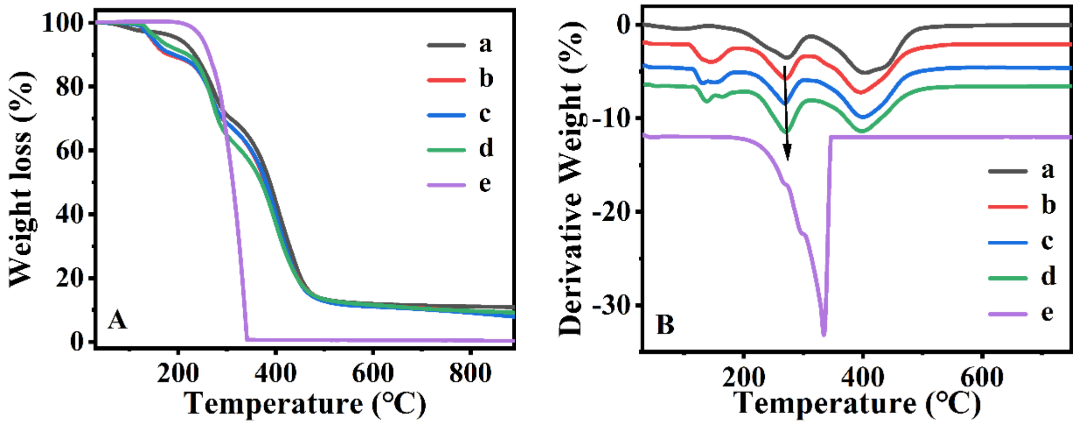

Figure S6. TG (A) and DTG (B) plots of (a) PAA, (b) P(NA-AA)-0-0.1, (c) P(NA-AA)-0-1, (d) P(NA-AA)-0-10, and (e) NA.

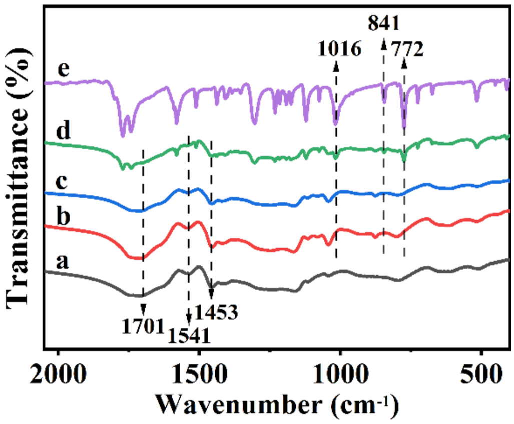

Figure S7. FT-IR spectra of (a) PAA, (b) P(NA-AA)-0-0.1, (c) P(NA-AA)-0-1, (d) P(NA-AA)-0-10, and (e) NA.

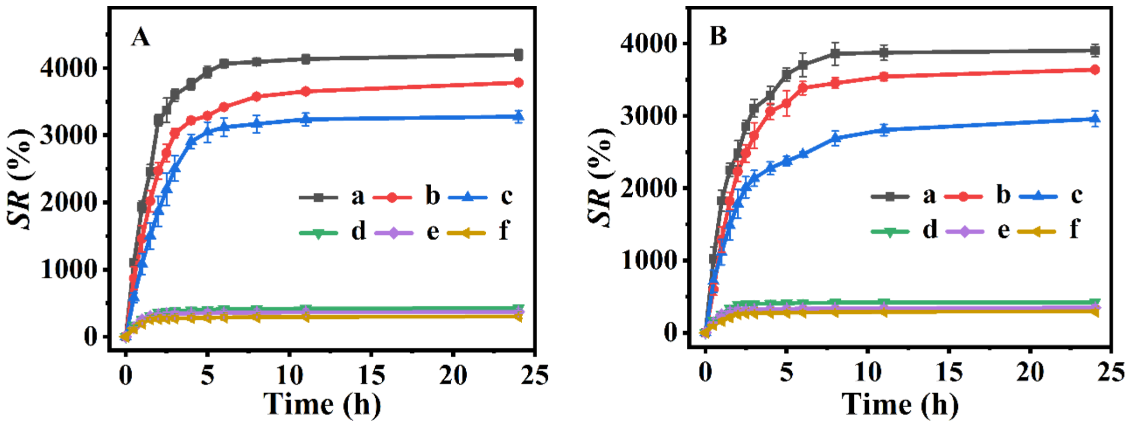

Figure S8. SR profiles of P(NA-AA)-1-y (A) and P(NA-AA)-7-y (B) at 37.0 °C under pH 7.4 (y = 0.1 (a), 1.0 (b), 10.0 (c)) and 2.0 (y = 0.1 (d), 1.0 (e), and 10.0 (f)).

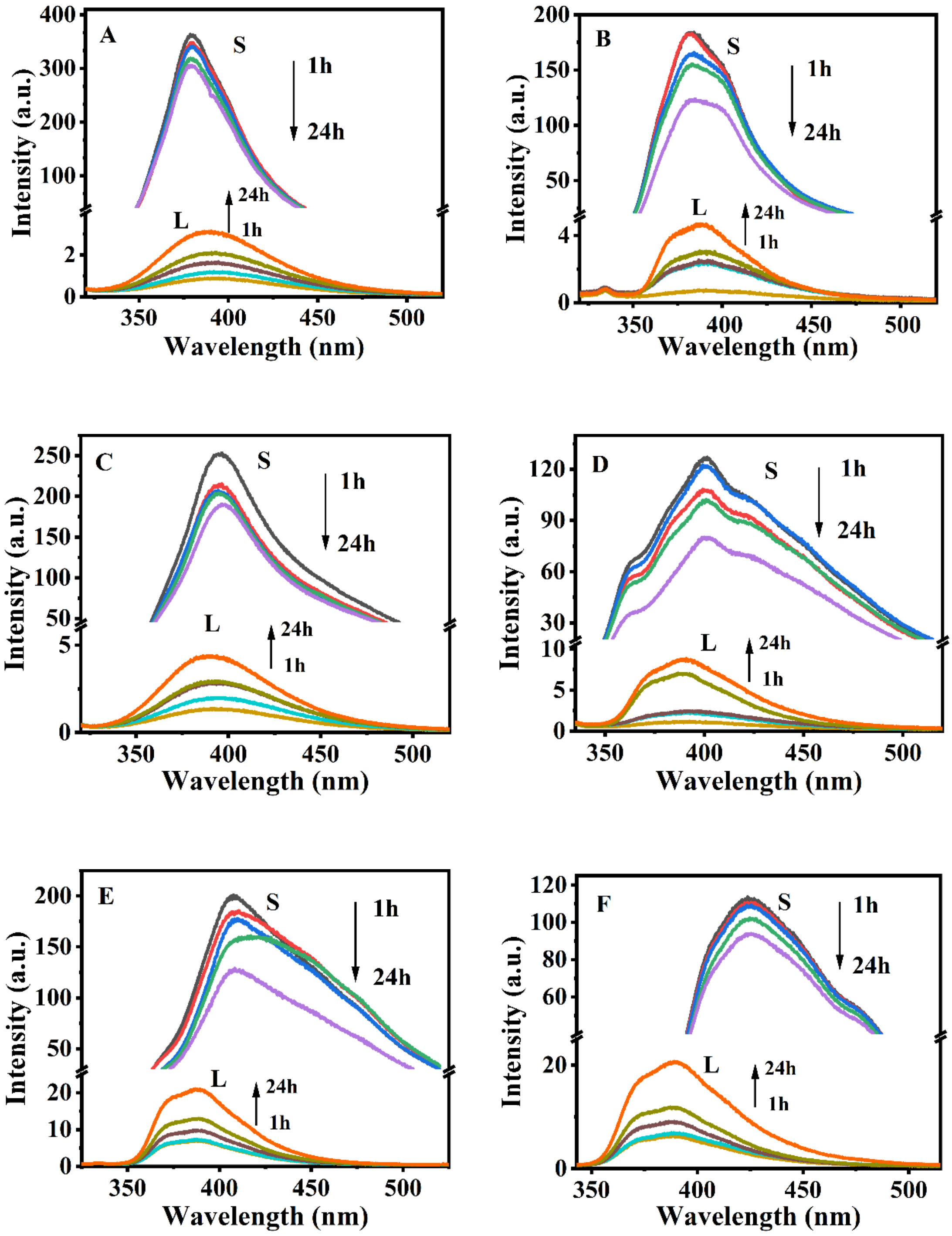

Figure S9. PL spectra of (A) P(NA-AA)-0-0.1, (B) P(NA-AA)-7-0.1, (C) P(NA-AA)-0-1, (D) P(NA-AA)-7-1, (E) P(NA-AA)-0-10, and (F) P(NA-AA)-7-10 with different swelling time under pH 7.4. Notes: S- represents the swollen solid and L- the filtrated solution obtained from the swelling system over time.

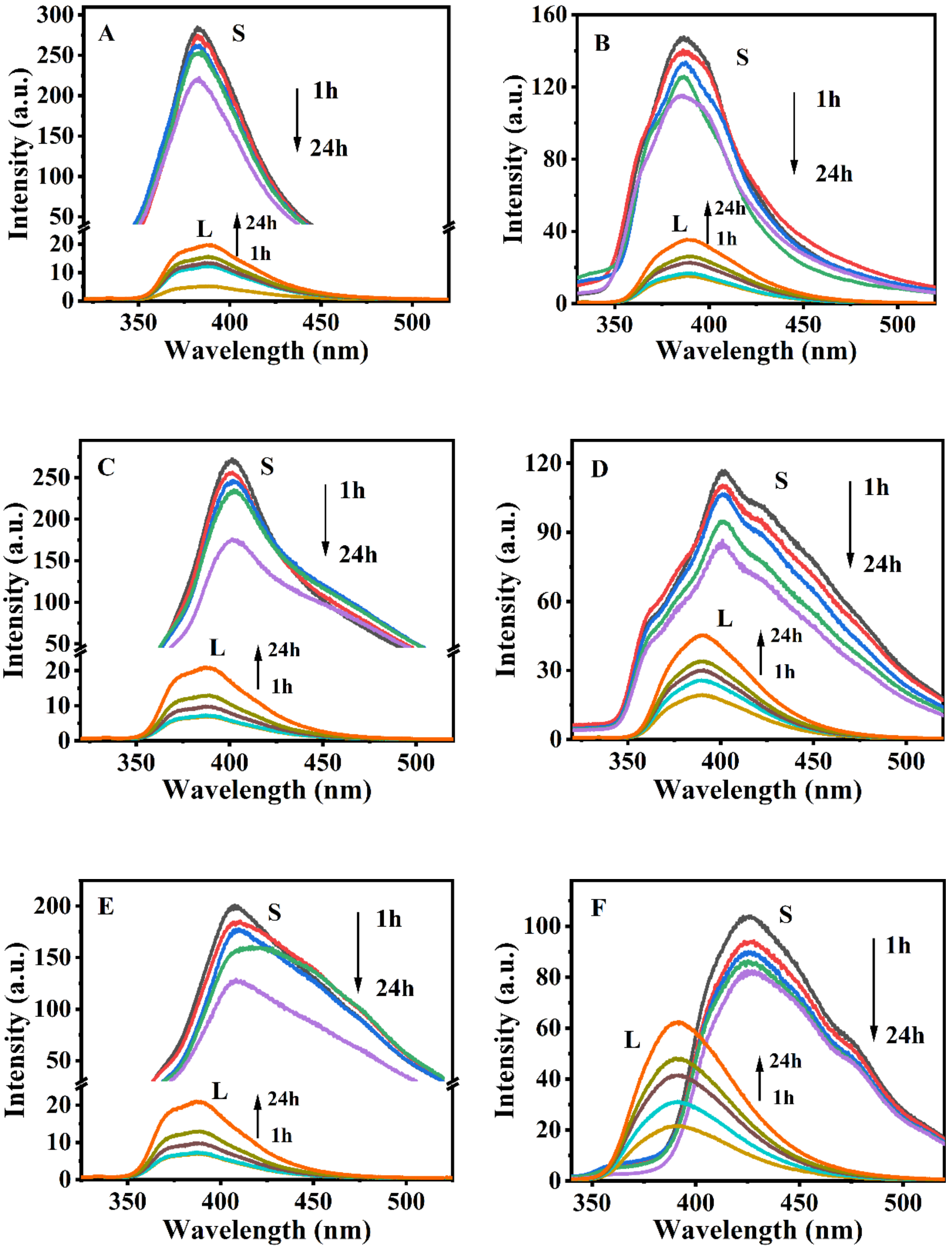

Figure S10. PL spectra of (A) P(NA-AA)-0-0.1, (B) P(NA-AA)-7-0.1, (C) P(NA-AA)-0-1, (D) P(NA-AA)-7-1, (E) P(NA-AA)-0-10, and (F) P(NA-AA)-7-10 with different shrinking time under pH 2.0.

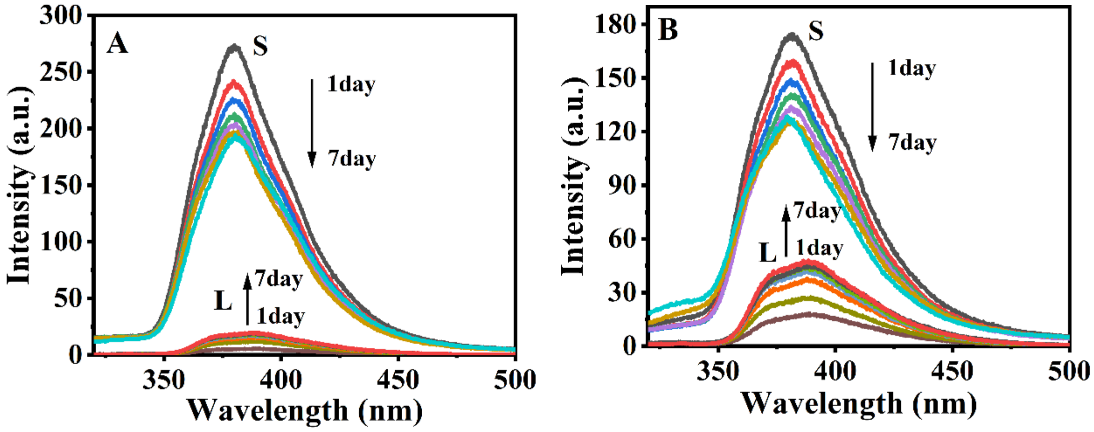

Figure S11. PL spectra of P(NA-AA)-0-0.1 with different swelling/shrinking time under pH 10.0 (A) and pH 1.0 (B).

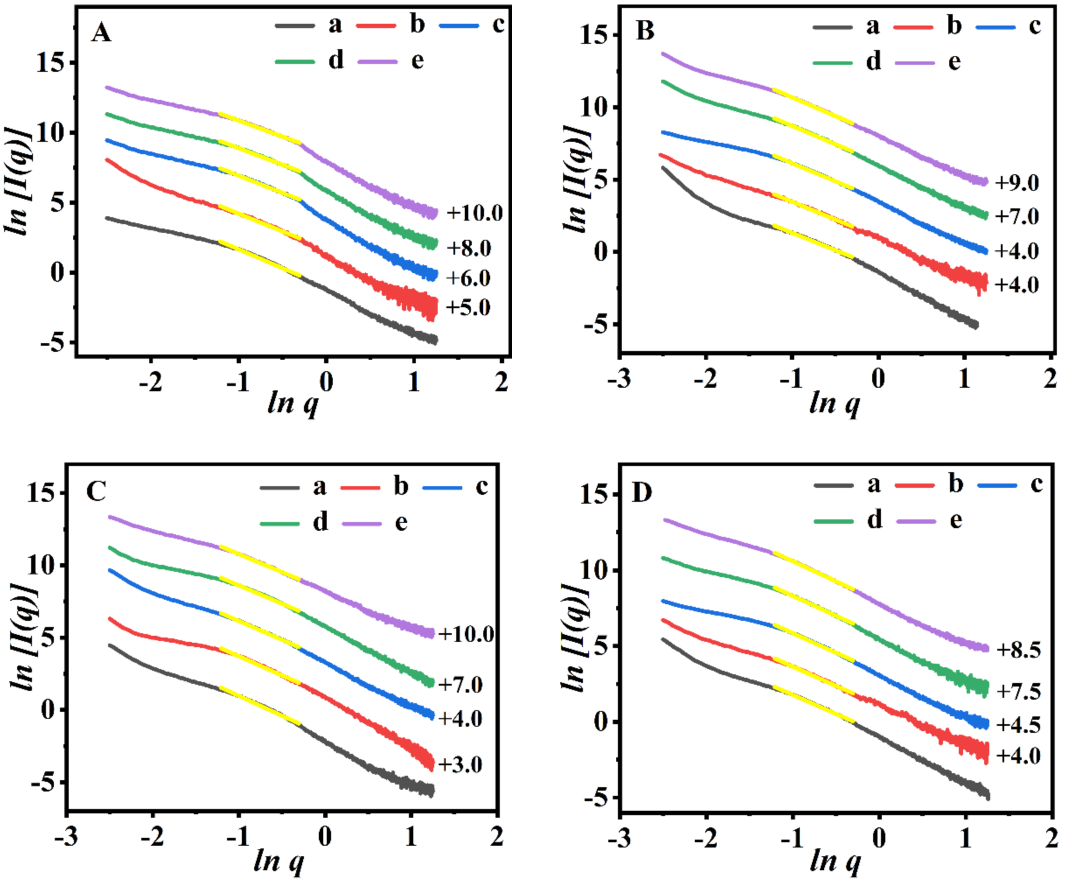

Figure S12. SAXS patterns (yellow lines were fitting curves based on the power low) of the swollen/shrunken (A and B) P(NA-AA)-1-0.1, and (C and D) P(NA-AA)-7-0.1 under pH 7.4 (A, and C) and 2.0 (B, and D) with the extended time. (a) 1 h, (b) 3 h, (c) 5 h, (d) 8 h, and (e) 24 h. The vertical offset values were presented in the Y-axis.

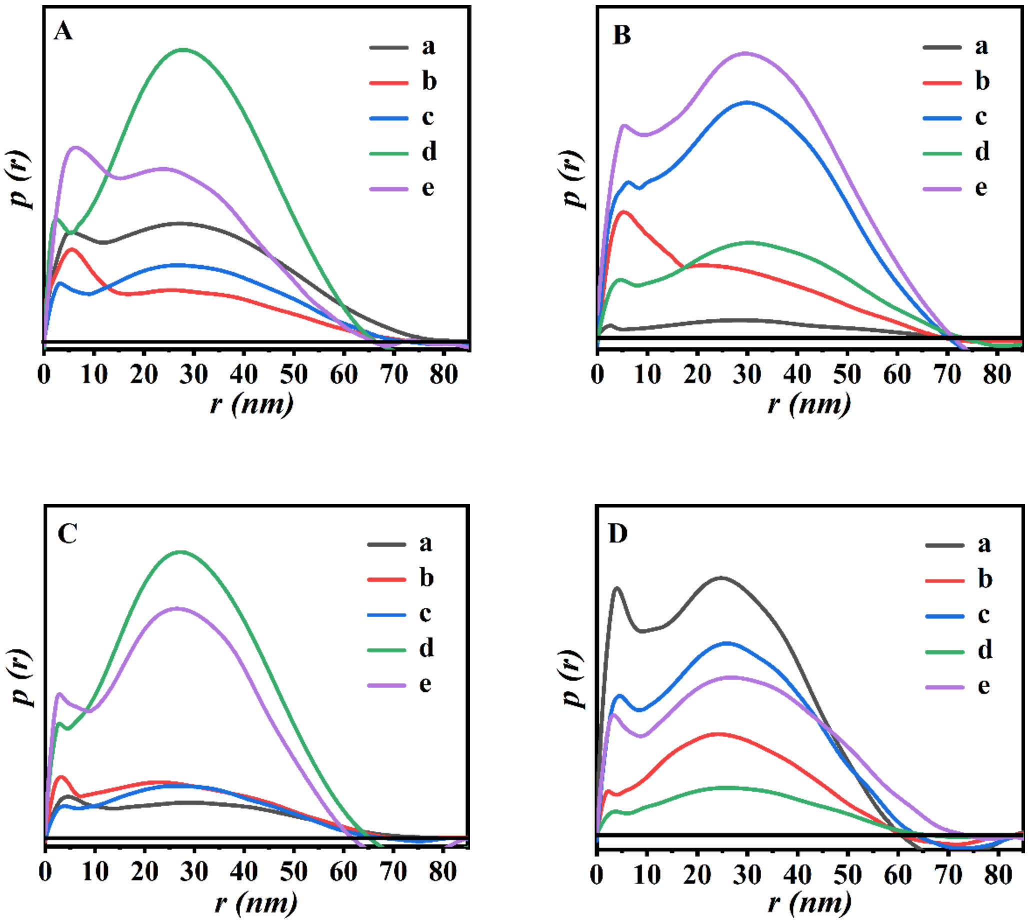

Figure S13. PDDF profiles of the swollen/shrunken P(NA-AA)-1-0.1 (A and C), and P(NA-AA)-7-0.1 (B and D) under pH 7.4 (A, and B) and 2.0 (D, and E) with the extended time. (a) 1 h, (b) 3 h, (c) 5 h, (d) 8 h, and (e) 24 h.

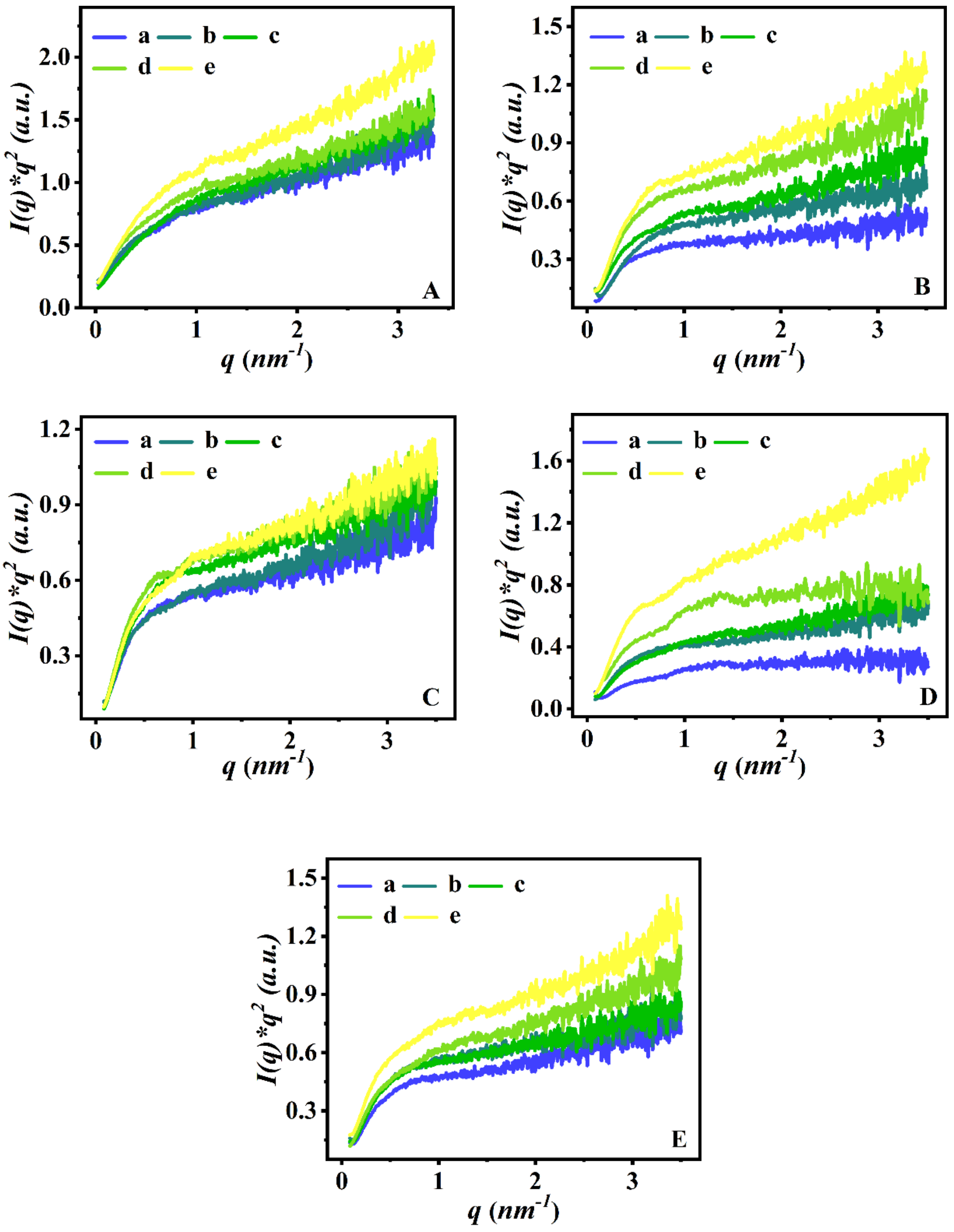

Figure S14. Kratky plots of the swollen P(NA-AA)-0-0.1 (A), P(NA-AA)-0-1 (B), P(NA-AA)-0-10 (C), P(NA-AA)-1-0.1 (D), and P(NA-AA)-7-0.1 (E) under pH 7.4 with the extension of the swelling time. (a) 1 h, (b) 3 h, (c) 5 h, (d) 8 h, and (e) 24 h.

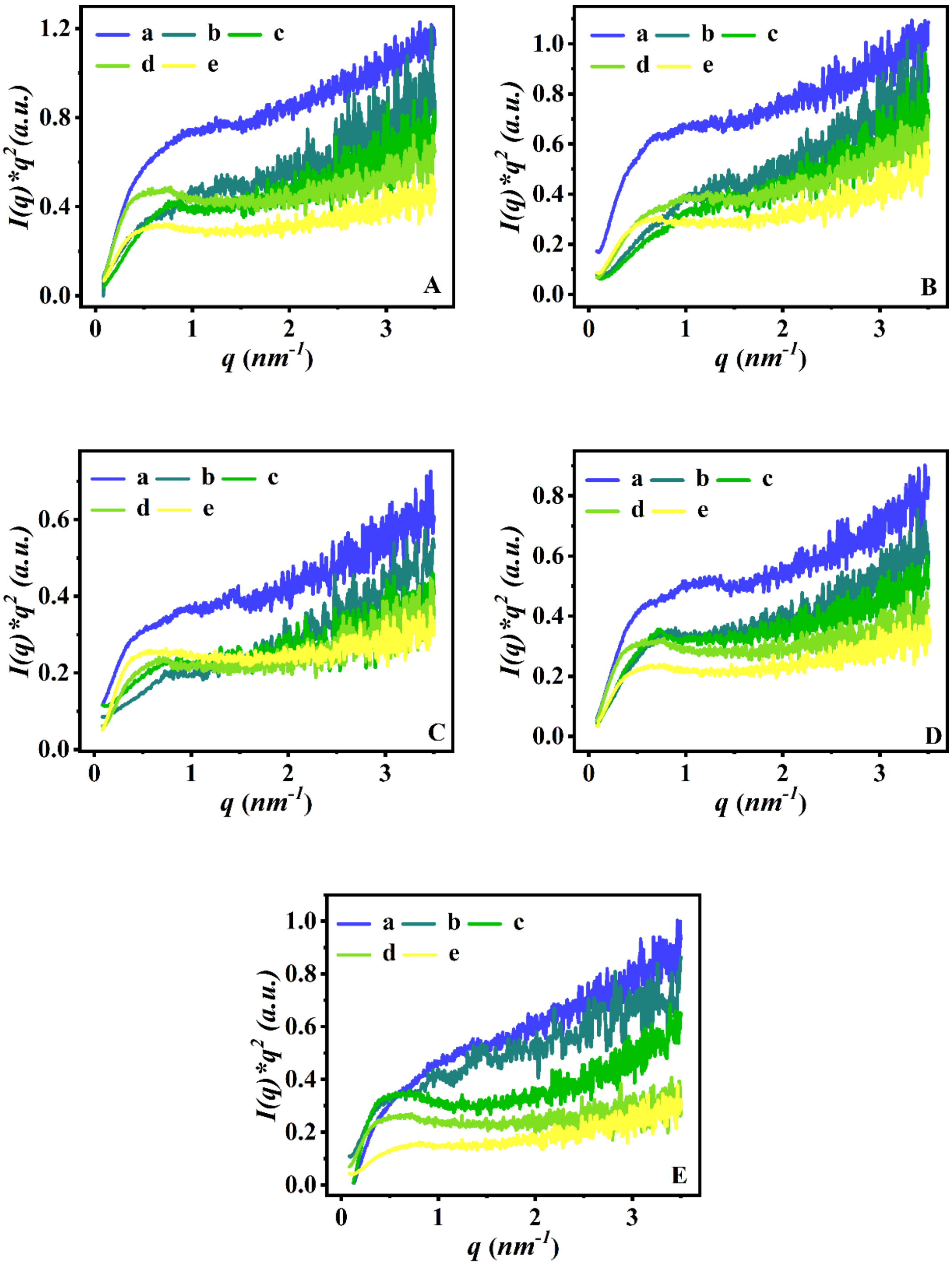

Figure S15. Kratky plots of the shrunken P(NA-AA)-0-0.1(A), P(NA-AA)-0-1 (B), P(NA-AA)-0-10 (C), P(NA-AA)-1-0.1 (D), and P(NA-AA)-7-0.1 (E) under pH 2.0 with the extension of the shrinking time. (a) 1 h, (b) 3 h, (c) 5 h, (d) 8 h, and (e) 24 h.

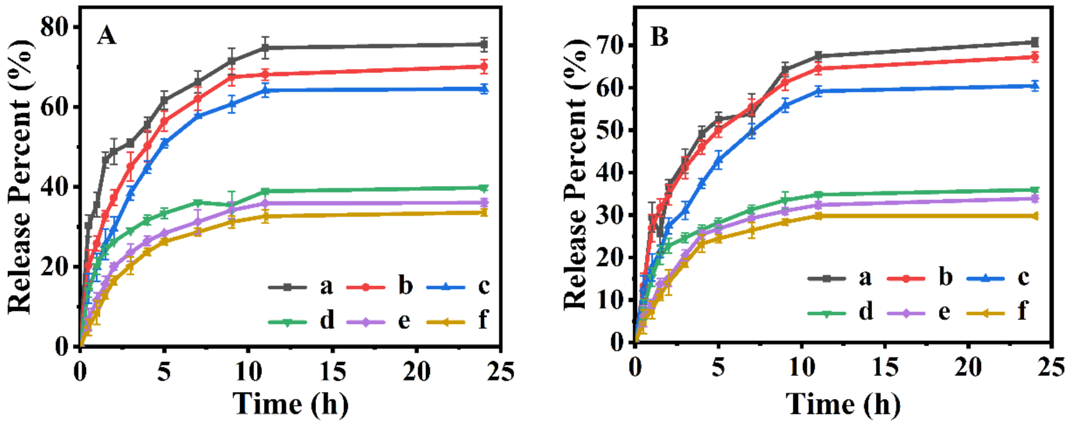

Figure S16. Cumulative IBU-releasing profiles from drug-loaded P(NA-AA)-1-y (A) and P(NA-AA)-7-y (b) at 37.0 °C under pH 2.0 (y = 0.1 (a), 1.0 (b), 10.0 (c)) and 7.4 (y = 0.1 (d), 1.0 (e), and 10.0 (f)).

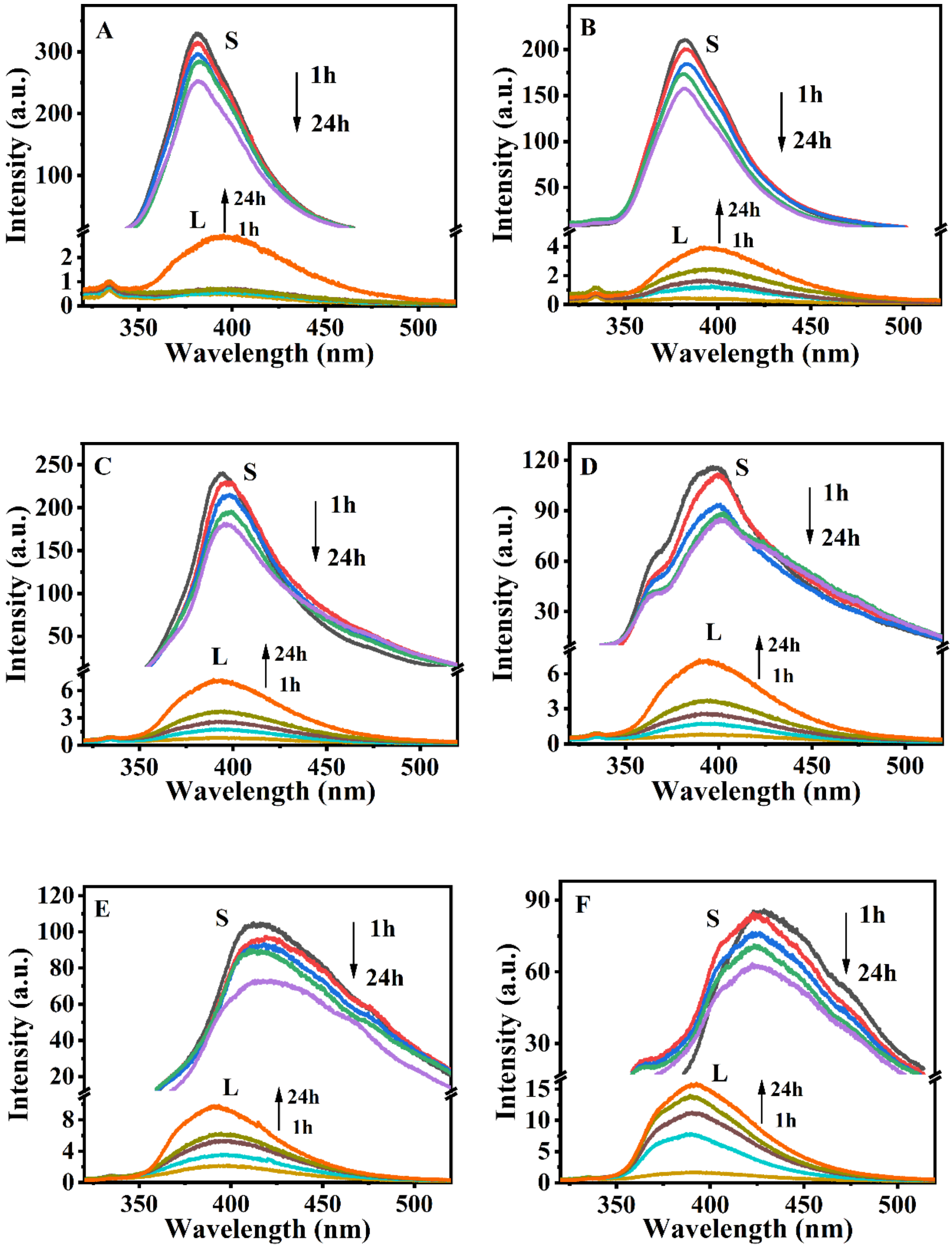

Figure S17. PL spectra of the drug-released (A) P(NA-AA)-0-0.1, (B) P(NA-AA)-7-0.1, (C) P(NA-AA)-0-1, (D) P(NA-AA)-7-1, (E) P(NA-AA)-0-10, and (F) P(NA-AA)-7-10 with different releasing time under pH 7.4. Notes: As described in experimental methods, the drug loaded samples were immersed in PBS system, 1.0 mL of releasing solution was withdrawn at a predetermined time. After filtered, the obtained solid was defined S, and corresponding filtrate liquid was defined L.

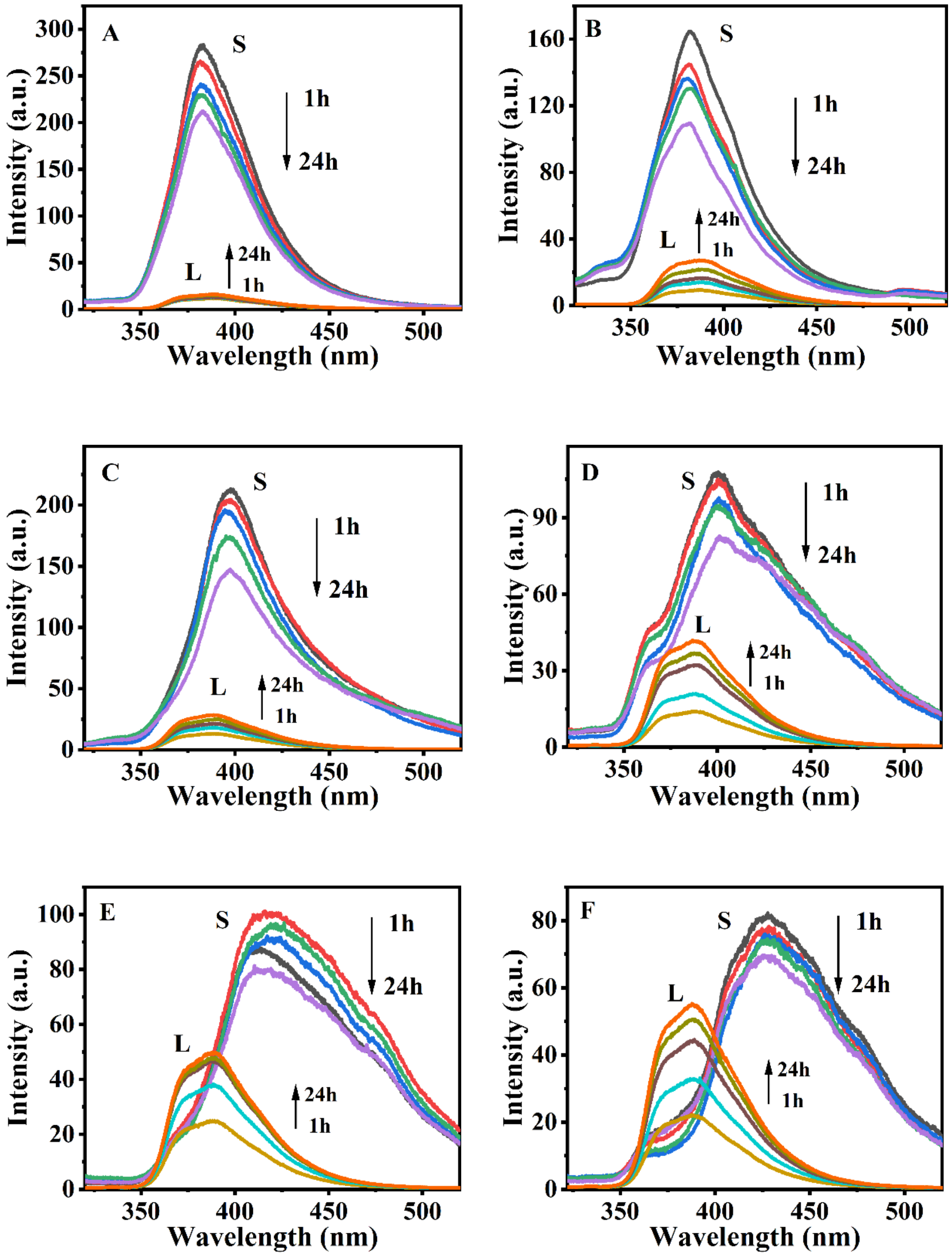

Figure S18. PL spectra of the drug-released (A) P(NA-AA)-0-0.1, (B) P(NA-AA)-7-0.1, (C) P(NA-AA)-0-1, (D) P(NA-AA)-7-1, (E) P(NA-AA)-0-10, and (F) P(NA-AA)-7-10 with different releasing time under pH 2.0.

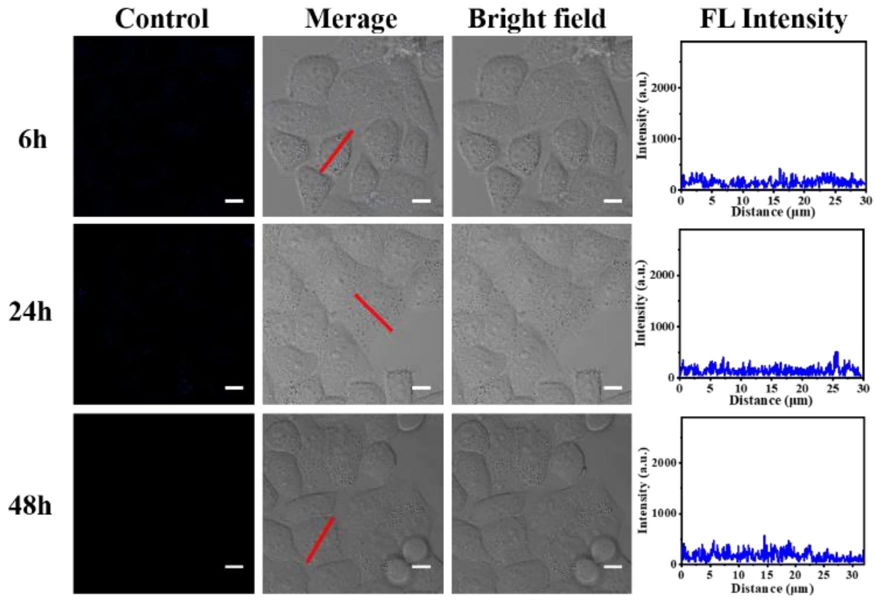

Figure S19. Time-dependent CLSM images of HeLa cells incubated as controlled samples. The scale bars are 10 μm.

Table S1. Summaries of P(NA-AA)-x-y drug-loaded capacity and cumulative release rate at pH 7.4 and pH 2.0.

Sample	IBU-Loaded capacity (%)	cumulative release rate (%)	
		pH 7.4	pH 2.0
P(NA-AA)-0-0.1	5.93	39.9	76.6
P(NA-AA)-0-1	5.47	36.5	72.6
P(NA-AA)-0-10	4.92	36.3	69.2
P(NA-AA)-1-0.1	5.66	39.8	75.6
P(NA-AA)-1-1	5.31	36.1	70.1
P(NA-AA)-1-10	4.38	33.6	64.5
P(NA-AA)-7-0.1	5.51	35.9	70.7
P(NA-AA)-7-1	5.17	33.9	67.2
P(NA-AA)-7-10	4.02	29.8	60.4
